# Neuroactive metabolites and bile acids are altered in extremely premature infants with brain injury

**DOI:** 10.1016/j.xcrm.2024.101480

**Published:** 2024-03-22

**Authors:** Manuel Pristner, Daniel Wasinger, David Seki, Katrin Klebermaß-Schrehof, Angelika Berger, David Berry, Lukas Wisgrill, Benedikt Warth

**Affiliations:** 1 Department of Food Chemistry and Toxicology, University of Vienna, 1090 Vienna, Austria; 2 Department of Pediatrics and Adolescent Medicine, Division of Neonatology, Pediatric Intensive Care and Neuropediatrics, Comprehensive Center for Pediatrics, Medical University of Vienna, 1090 Vienna, Austria; 3 Center for Microbiology and Environmental Systems Science, Department of Microbiology and Ecosystem Science, Division of Microbial Ecology, University of Vienna, 1090 Vienna, Austria; 4 Joint Microbiome Facility of the Medical University of Vienna and the University of Vienna, 1090 Vienna, Austria

**Keywords:** extremely premature infant, untargeted metabolomics, bile acid amino acid conjugates, gut-immune-brain axis, neonatal brain injury

## Abstract

The gut microbiome is associated with pathological neurophysiological evolvement in extremely premature infants suffering from brain injury. The exact underlying mechanism and its associated metabolic signatures in infants are not fully understood. To decipher metabolite profiles linked to neonatal brain injury, we investigate the fecal and plasma metabolome of samples obtained from a cohort of 51 extremely premature infants at several time points, using liquid chromatography (LC)-high-resolution mass spectrometry (MS)-based untargeted metabolomics and LC-MS/MS-based targeted analysis for investigating bile acids and amidated bile acid conjugates. The data are integrated with 16S rRNA gene amplicon gut microbiome profiles as well as patient cytokine, growth factor, and T cell profiles. We find an early onset of differentiation in neuroactive metabolites between infants with and without brain injury. We detect several bacterially derived bile acid amino acid conjugates in plasma and feces. These results provide insights into the early-life metabolome of extremely premature infants.

## Highlights

• Metabolic differentiation is already initiated in the first week of life
• Early metabolic alterations involve neuroactive and neurotoxic compounds
• Several recently discovered bile acid amino acid conjugates are detected
• Changed abundance of heme metabolites, bile acids, and steroid sulfates is observed

## Graphical abstract

**Figure:**
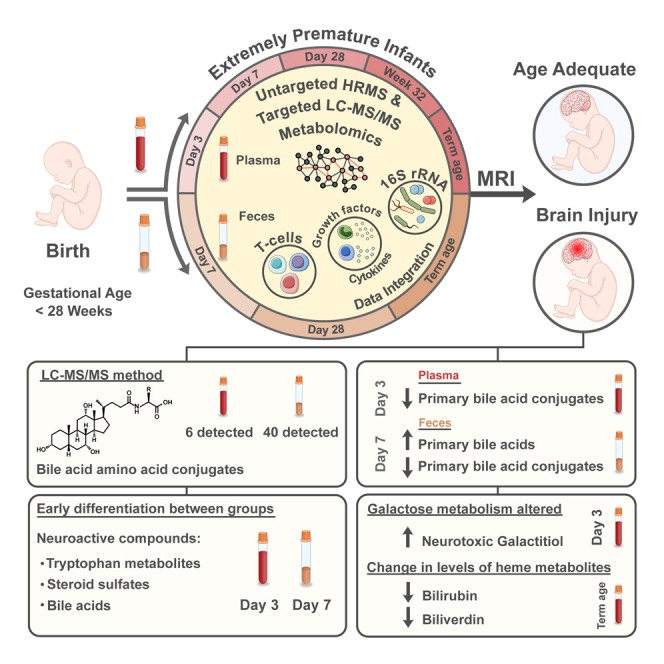

## Teaser

Pristner et al. use untargeted and targeted metabolomics to investigate the plasma and fecal metabolome of extremely premature infants with brain injury and the correlation with gut microbiome and immunological data. The results show an early differentiation in neuroactive metabolites and bile acids in infants diagnosed with brain injury later in life.

## Introduction

Extremely premature infants are born before 28 weeks of gestation and represent a highly vulnerable patient cohort. While the overall survival rate of premature infants has improved in the last decades, a significant number of infants still suffer from severe morbidities and life-long neurodevelopmental impairment.^1^ Extremely premature infants are born during a critical window of human fetal development, leaving them prone to early physical, microbial, and environmental exposures. Within this third-trimester window, neurophysiological maturation and development appears to be highly influenced by postnatal factors, especially by the developing gut microbiome. We recently described gut-immune-brain interactions in a clinical cohort of 60 extremely premature infants and found that *Klebsiella* overgrowth is associated with pro-inflammatory immunological imprinting and neonatal brain injury.^2^ Therefore, early gut-immune-mediated and host-microbe signaling might have implications for early development.^3^

The gut-immune-brain axis represents a complex bidirectional communication system composed of various neural pathways with neuropeptides, cytokines, and hormones as signaling molecules. An important communication pathway between gut microbiota and the central nervous system (CNS) is the enteric nervous system.^4^ Recent advances in understanding the gut-immune-brain axis have shown that microbes play a significant role in signal transduction, which, in return, can modulate the physiology of the host.^5^^,^^6^ With host-affecting bacterial metabolites such as bile acid metabolites, short-chain fatty acids, amino acid metabolites, serotonin, and γ-aminobutyric acid (GABA),^7^ gut bacteria have been associated with functions and diseases of the CNS.^8^^,^^9^ The synthesis of phenylalanine, tryptophan (TRP), and tyrosine via the shikimate pathway and subsequent catabolism results in formation of neurotransmitters L-3,4-dihydroxyphenylalanine, dopamine, epinephrine, and norepinephrine.^8^ TRP-derived indole and its derivatives indoleacetic acid, indolepropionic acid, and tryptamine induce neuroactivity and potentially limit TRP availability for the neurotransmitter synthesis.^10^

Recently, it has been shown that the communication between the bile system and gut microbiota is bidirectional in nature, potentially involving neural modulation. In humans, *de novo* synthesized primary bile acids are cholic acid (CA) and chenodeoxycholic acid (CDCA). Upon performing their role in lipid digestion,^11^ most bile acids are recycled back to the liver via the enterohepatic cycle. Various members of the gut microbiota express bile salt hydrolase enzymes that deconjugate bile acids from taurine and glycine, which reduces the efficiency of their reabsorption in the enterohepatic cycle.^12^^,^^13^ Deconjugation and dehydroxylation, oxidation, and epimerization of bile acids by gut microbiota are the main mechanisms for the formation of secondary bile acids such as deoxycholic acid (DCA), lithocholic acid (LCA), hyodeoxycholic acid (HDCA), and ursodeoxycholic acid (UDCA).^14^ These acids can be absorbed into the colon by passive diffusion and transported to the liver, where a repeated conjugation with glycine and taurine can occur, followed by secretion into the intestinal tract.^15^ A particularly important role of bile acids is their relation to inflammatory responses. While hydrophobic bile acids may act as pro-inflammatory signals, hydrophilic bile acids were shown to have the opposite effect. Moreover, deficits in luminal levels of bile acids have been correlated with gut bacterial overgrowth, intestinal inflammation, and tissue damage.^16^^,^^17^ Recent evidence suggested that bile acids might directly affect the brain.^18^^,^^19^

The investigation of the diverse group of chemical compounds affecting the brain-gut axis poses a methodological challenge and requires a comprehensive analytical approach. High-resolution mass spectrometry (HRMS)-based untargeted metabolomics is an emerging and powerful approach that enables the global analysis of endogenous and exogenous metabolites, thereby revealing information about progression, causes, and potential biomarkers of diseases.^20^^,^^21^ Metabolomics investigations of extremely premature infants have thus far been limited to only a few publications investigating only a single sample matrix or using a less comprehensive approach.^22^^,^^23^^,^^24^^,^^25^

Based on the previously found association of the overgrowth of *Klebsiella* with pro-inflammatory immunological imprinting and neonatal brain injury,^2^ an investigation of the blood and fecal metabolome was performed as the next step for generating insights into the possible molecular mechanisms underlying brain injury and for deciphering potential links to the gut microbiome. As a conclusive diagnosis of brain injury by magnetic resonance imaging (MRI) can only be done at a later developmental stage (term-equivalent age), there is also a need for early metabolic indicators for the later occurrence of brain injury. A deeper knowledge about underlying mechanisms and early metabolic predictors might pave the way for the development of an early intervention strategy to stop the formation of brain injury or at least reduce the degree of neurological damage. Here, we investigated the fecal and plasma metabolome in samples from a cohort^2^ of extremely premature infants collected at several time points by untargeted liquid chromatography (LC)-HRMS and targeted LC-tandem mass spectrometry (LC-MS/MS) based metabolomics. The resulting data were integrated with 16S rRNA gene amplicon gut microbiome profiles as well as patient cytokine, growth factor, and T cell profiles.

## Results

From a cohort of 51 extremely premature infants hospitalized in the same hospital, 120 plasma samples at five time points (day 3, day 7, day 28, correction week 32, and term-equivalent age) and 75 fecal samples at three time points (day 7, day 28, and term-equivalent age) were analyzed. The cohort consisted of two patient groups, one with age-adequate findings or mild brain injuries diagnosed by cranial MRI at term-equivalent age, named the control group (CTR, n = 36), and one group with pathological and severe brain injuries, named the pathological group (PAT, n = 15).

**Figure 1:**
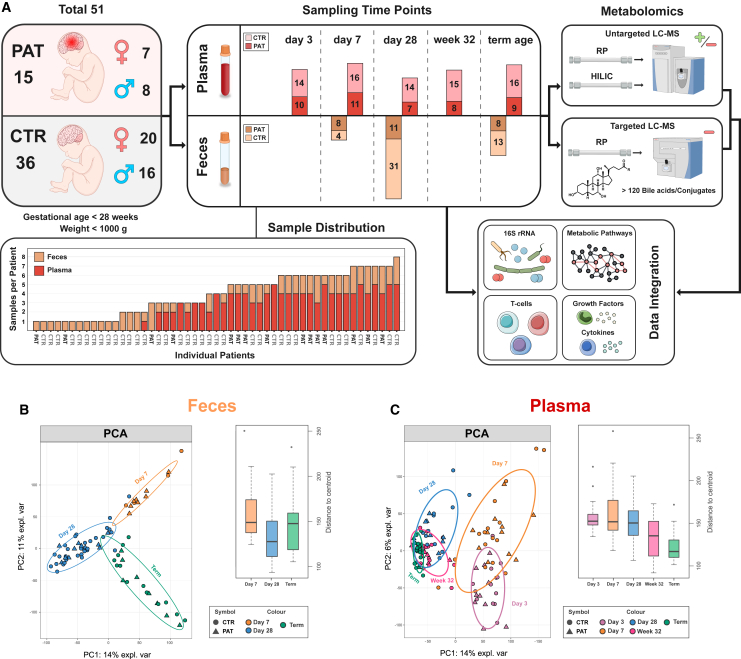
Study design and metabolomic differences over time (A) Visualization of the study design. (B) PCA of untargeted metabolomics dataset and respective distances to the centroid for (B) feces and (C) plasma.

A visual summary of the patient cohort, sample composition, and experiments is illustrated in Figure 1A. Additional information about the patient cohort, sampling procedures, and details of previous T cell, cytokine, growth factor, and 16S rRNA gene amplicon analysis, and determination of brain damage, is described in detail in the supplemental information and our previous publication.^2^ Table 1 presents an overview of the subcohort used for this study, including the statistical significance of associations between variables and experimental groups. Retinopathy of prematurity (ROP) was observed in all infants of the pathological group and exhibited a significant relationship with the experimental groups (Table 1). A significant association between mode of delivery, with fewer C-sections in the pathological group, was also observed but was not statistically significant after multiple testing correction in the subgroup of infants. An in-depth discussion of these findings can be found in our previous publication.^2^

**Table 1:** Overview of the cohort of extremely premature infants

Subject cohort demographics				
Variable	CTR	PAT	Fisher’s exact test/Student’s t test p value	Bonferroni corrected p value
No. of infants	36	15	–	–
Birth date (quarter)	–	–	0.336	1
Birth date (month)	–	–	0.294	1
Sex assigned at birth (% female)	56	47	0.759	1
BPD (%)	25	47	0.187	1
ROP (%)	42	100	<0.001	0.002
PDA (%)	31	40	0.532	1
NEC (%)	8	0	0.545	1
Maternal antibiotics (%)	50	60	0.536	1
C-section (%)	86	46	0.011	0.264
Connatal infection (%)	36	53	0.734	1
Antibiotic intervention (%)	72	87	0.470	
pPROM (%)	56	47	0.75	1
Gestational age (weeks)	25.6 ± 1.2	24.9 ± 1.4	0.137	1
Birth weight (g)	774.4 ± 138.6	740.3 ± 168.8	0.497	1
Birth length (cm)	33.1 ± 2.2	32.8 ± 2.3	0.635	1
Birth head circumference (cm)	23.2 ± 1.5	23 ± 1.9	0.621	1
Weight slope	0.016 ± 0.001	0.015 ± 0.002	0.225	1
Apgar score at 1 min	7.3 ± 0.8	7.1 ± 1.5	0.761	1
Apgar score at 5 min	8.5 ± 0.6	8.3 ± 1.3	0.436	1
Apgar score at 10 min	8.9 ± 0.4	8.9 ± 0.4	0.868	1
Hospitalization time (days)	88.2 ± 23.3	96.6 ± 29.5	0.605	1
Antibiotic intervention (days)	8.7 ± 5.9	9.9 ± 8.7	0.662	1
Age at discharge (weeks)	38.2 ± 3.1	38.9 ± 3.2	0.780	1
Discharge head circumference (cm)	32.7 ± 2.5	33.1 ± 2.8	0.979	1
Discharge length (cm)	46.2 ± 3.9	46.3 ± 3.7	0.716	1
Discharge weight (kg)	2.9 ± 0.8	2.8 ± 0.5	0.420	1
O_2 max_ at birth (%)	66.7 ± 21.1	57.3 ± 22.4	0.180	1

CTR, control group; PAT, pathological group; pPROM, preterm premature rupture of membranes; BPD, bronchopulmonary dysplasia; ROP, retinopathy of prematurity; PDA, persistent ductus arteriosus; NEC, necrotizing enterocolitis. Data are presented as mean ± SD or percentage.

### Development of the plasma and fecal metabolome of extremely premature infants over time

For characterization of the gut and plasma metabolome, each sample was analyzed by LC-HRMS in an untargeted manner using two complementary separation methods, reversed-phase and hydrophilic interaction chromatography, in positive and negative polarity mode to ensure the coverage of a broad spectrum of compounds and chemical classes. For a general overview of the differences between the groups over time, a principal component analysis (PCA) was performed on the combined data of the untargeted LC-HRMS experiments.

This was followed by a permutational multivariate analysis of variance (PERMANOVA), which found that time point was a significant factor (plasma, p < 0.001; feces, p < 0.001; variation explained by experimental group: plasma = 21%, feces = 19%), while disease diagnosis was not significant. The experimental group explained in plasma <1% and in feces <1% of PERMANOVA variation, while patient ID explained 30% in plasma and 55% in feces. In addition, the dispersion of samples at the different sampling time points in plasma (average Euclidean distance to centroid: day 3 = 157.0, day 7 = 164.5, day 28 = 151.8, week 32 = 133.6, term = 122.6) (Figure 1C) and feces (average Euclidean distance to centroid: day 7 = 160.1, day 28 = 132.9, term = 146.1) (Figure 1B) was analyzed. The difference in dispersion based on time point was statistically significant by ANOVA in both plasma and feces (plasma, p < 0.001; feces, p = 0.017).

### Metabolic changes from a pathway perspective

**Figure 2:**
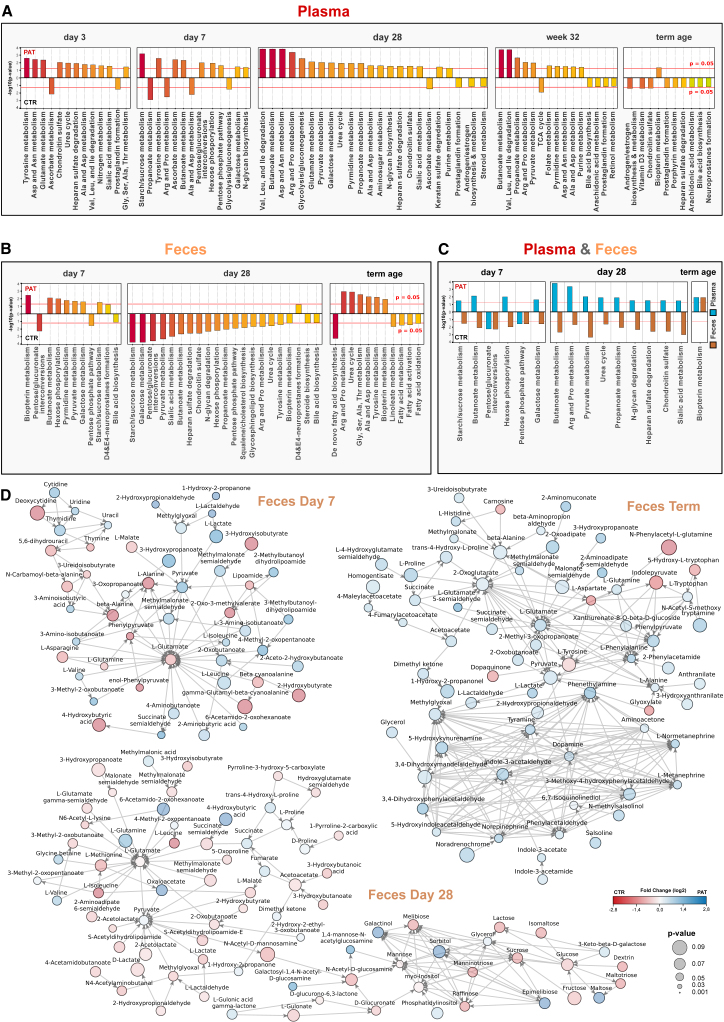
Observed metabolic changes at the pathway level (A–C) Enriched metabolic pathways and their combined p values for (A) plasma, (B) feces, and (C) their intersection. (D) Activity networks of feces created by mummichog.

To put the untargeted LC-HRMS experiments into biological context, we used a combination of mummichog,^26^ a tool for pathway enrichment based on putative compound annotation, and a gene set enrichment analysis-related approach (Figures 2A–2C). In Figure 2 the altered metabolic pathways in plasma (Figure 2A), feces (Figure 2B), and the intersection between plasma and feces (Figure 2C) can be seen. Pathway enrichment analysis is restricted to the predefined human genome-scale metabolic model, and its application for the analysis of microbial pathways is limited. Therefore, we also created activity networks for the fecal samples using the Python package of mummichog (Figures 2D–2F).

#### Plasma

The metabolism of several amino acids was significantly modulated in plasma at day 3, day 7, and day 28, including the metabolism of glutamate, an important neurotransmitter, at day 3 and day 28 (day 3: p = 0.003; day 28: p = 0.007). Also, pathways involved in carbohydrate metabolism were significantly altered at day 7 and to a lesser extent at day 28.

#### Feces

At all three time points, a significant or near-significant enrichment was found in biopterin metabolism (day 7: p = 0.004; day 28: p = 0.055; term: p = 0.010). Various pathways involved in carbohydrate metabolism were significantly modulated both at day 7 and day 28 in the feces. The pathway of the urea cycle was also significantly affected at both day 28 and term-equivalent age (day 28: p = 0.023; term: p = 0.001) in feces. Tyrosine metabolism, connected to the synthesis of the neurotransmitter dopamine, was significantly altered at day 28 and term-equivalent age in feces (day 28: p = 0.034; term: p = 0.005). Biopterin metabolism was affected significantly in the feces at each sampling time point. In the activity networks of the feces (Figure 2D), amino acids together with pyruvate and their related metabolites were the most prominent and well-connected metabolites at all three time points.

### Metabolic profiles in plasma are already differentiated 3 days after delivery

**Figure 3:**
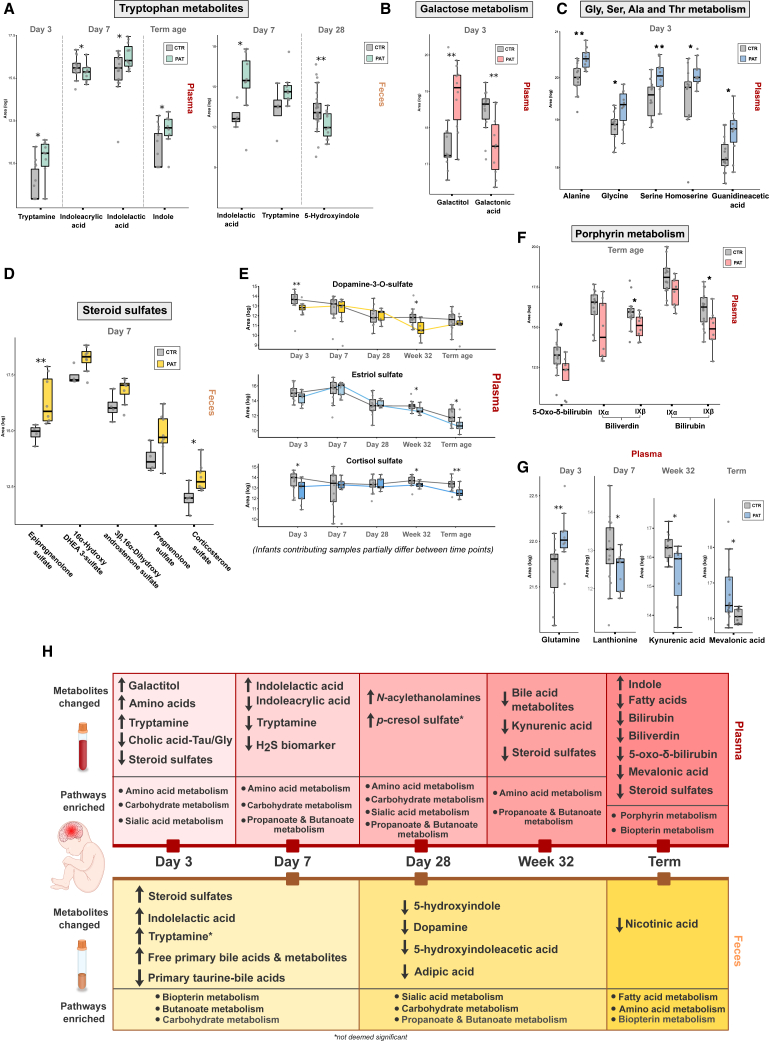
Selected metabolites altered between groups at different time points in plasma and feces (A–G) Box plots of selected metabolites. Plasma: day 3: n_CTR_ = 14, n_PAT_ = 10; day 7: n_CTR_ = 16, n_PAT_ = 11; day 28: n_CTR_ = 14, n_PAT_ = 7; week 32: n_CTR_ = 15, n_PAT_ = 8; term age: n_CTR_ = 16, n_PAT_ = 9. Feces: day 7: n_CTR_ = 4, n_PAT_ = 8; day 28: n_CTR_ = 31, n_PAT_ = 11. ∗p < 0.05, ∗∗p < 0.01, ∗∗∗p < 0.001. (H) Selected overview of differentiated metabolites and pathways between groups over all time points. Arrows pointing up indicate an increase in the pathological group.

Plasma samples were investigated by untargeted metabolomics followed by evaluation in a purely untargeted fashion combined with a targeted suspect screening for compounds of interest. A positive fold change indicates an increase in the pathological group (Figure 3).

#### Day 3

The untargeted evaluation of the metabolomics data revealed 112 significantly altered features (identification confidence level 2 [ICL-2]: 1; ICL-3: 17; ICL-4: 19; unidentified: 75) with a log_2_ fold change (FC) greater than |2|. The metabolites with the highest discriminatory power between the experimental groups and highest annotation confidence consisted of the bile acid conjugate tauro-1-hydroxycholic acid (ICL = 3, score = 0.88, p = 0.006, FC-log_2_ = 3.1), the steroid sulfate 11,20-dioxo-5b-pregnane-3-α,17,21-triol-3-sulfate (ICL = 3, score = 0.79, p = 0.026, FC-log_2_ = −2.2), hydroxyphenyllactic acid (ICL = 3, score = 0.99, p = 0.049, FC-log_2_ = 3.6), and 4-hydroxycinnamic acid (ICL = 3, score = 0.99, p = 0.024, FC-log_2_ = 4.1) and its glycoside 1-*O*-*p*-coumaroyl-β-D-glucose (ICL = 3, score = 0.79, p = 0.003, FC-log_2_ = 3.1). During the suspect screening, 26 metabolites (ICL-1: 15; ICL-3: 11) were significantly changed, including, among others, the amino acids alanine (ICL = 1, p = 0.001, FC-log_2_ = 0.6), glutamine (ICL = 1, p = 0.002, FC-log_2_ = 0.5), asparagine (ICL = 1, p = 0.005, FC-log_2_ = 0.9), serine (ICL = 1, p = 0.007, FC-log_2_ = 0.7), homoserine (ICL = 1, p = 0.042, FC-log_2_ = 0.6), citrulline (ICL = 1, p = 0.042, FC-log_2_ = 0.8), and sarcosine (ICL = 1, p = 0.048, FC-log_2_ = 1.3), glyoxylic acid (ICL = 1, p = 0.048, FC-log_2_ = 0.7), the TRP metabolite tryptamine (ICL = 1, p = 0.012, FC-log_2_ = 2.7), the metabolites of galactose metabolism galactitol (ICL = 1, p = 0.006, FC-log_2_ = 1.4) and galactonic acid (ICL = 3, score = 0.99, p = 0.007, FC-log_2_ = −1.1), and the sulfated compounds 19-nortestosterone sulfate (ICL = 3, score = 0.75, p = 0.002, FC-log_2_ = −1.2), dopamine 3-*O*-sulfate (ICL = 3, score = 0.78, p = 0.002, FC-log_2_ = −1.3), 3-β,16α-dihydroxyandrostenone sulfate (ICL = 3, score = 0.85, p = 0.026, FC-log_2_ = −1.1), and 3,5-tetrahydroaldosterone sulfate (ICL = 3, score = 0.76, p = 0.007, FC-log_2_ = −1.0).

#### Day 7

Overall, 12 significantly altered features (ICL-4: 2; unidentified: 10) with a log_2_ FC greater than |2| were observed during the untargeted evaluation, and four metabolites (ICL-1: 1; ICL-2: 1; ICL-3: 2) were significantly changed in the scope of the targeted evaluation. The significant metabolites consisted of lanthionine (ICL = 3, score = 0.82, p = 0.039, FC-log_2_ = −1.1) and the TRP metabolites tryptamine (ICL = 1, p = 0.048, FC-log_2_ = −1.4), indoleacrylic acid (ICL = 3, score = 0.83, p = 0.044, FC-log_2_ = 1.1), and indolelactic acid (ICL = 3, score = 0.87, p = 0.044, FC-log_2_ = 1.0).

#### Day 28

The significantly changed features with a log_2_ FC greater than |2| consisted of 39 features (ICL-2: 4; ICL-3: 8; ICL-4: 6; unidentified: 21) for the untargeted evaluation. The metabolite group with the highest distinction between the experimental groups and highest annotation confidence was *N*-alkylated ethanolamines, including *N*-decyl-ethanolamine (ICL = 3, score = 0.79, p < 0.001, FC-log_2_ = 2.5), *N*-caprylyl-diethanolamine (ICL = 3, score = 0.81, p = 0.001, FC-log_2_ = 5.2), *N*-dodecanoyl-ethanolamine (ICL = 3, score = 0.75, p = 0.005, FC-log_2_ = 4.8), *N*-decanoyl-ethanolamine (ICL = 3, score = 0.76, p = 0.009, FC-log_2_ = 3.0), and *N*-octyl-ethanolamine (ICL = 3, score = 0.86, p = 0.002, FC-log_2_ = 3.1). During the targeted evaluation, only *N*-acetylcitrulline (ICL = 3, score = 0.75, p = 0.024, FC-log_2_ = −1.6) was regarded as significant. In addition, elevated levels of *p*-cresol sulfate were observed in the pathological group (ICL = 1, p = 0.067, FC-log_2_ = 1.1), although not deemed statistically significant based on p value.

#### Week 32

The untargeted evaluation of the metabolomics data for plasma at week 32 revealed 72 significantly altered features (ICL-2: 1; ICL-3: 9; ICL-4: 10; unidentified: 52) with a log_2_ FC greater than |2|. The metabolites with the highest discriminatory power between the experimental groups and highest annotation confidence consisted of the bile acids glycochenodeoxycholyl-7-sulfate (ICL = 3, score = 0.79, p = 0.007, FC-log_2_ = −8.0), 7-oxoglycodeoxycholate (ICL = 3, score = 0.83, p = 0.004, FC-log_2_ = −2.9), and 7-oxotaurodeoxycholate (ICL = 3, score = 0.93, p = 0.004, FC-log_2_ = −2.5). For the suspect screening, six metabolites (ICL-1: 3; ICL-3: 3) were significantly altered between groups, consisting of spermidine (ICL = 1, p = 0.047, FC-log_2_ = 1.7), kynurenic acid (ICL = 1, p = 0.032, FC-log_2_ = −1.0), isocitrate (ICL = 1, p = 0.013, FC-log_2_ = 0.7), and the sulfated compounds dopamine 3-*O*-sulfate (ICL = 3, score = 0.78, p = 0.013, FC-log_2_ = −1.8), estriol 3-sulfate (ICL = 3, score = 0.80, p = 0.046, FC-log_2_ = −0.9), and cortisol 21-sulfate (ICL = 3, score = 0.96, p = 0.021, FC-log_2_ = −0.6).

#### Term-equivalent age

At term-equivalent age, 170 features (ICL-3: 22; ICL-4: 29; unidentified: 119) with a log_2_ FC greater than |2| were significantly altered during the untargeted evaluation. The most significantly altered metabolites with high annotation confidence were 3-carboxy-4-methyl-5-propyl-2-furanpropionic acid (ICL = 3, score = 0.99, p = 0.008, FC-log_2_ = −4.6) and 1-methyluric acid (ICL = 3, score = 0.99, p = 0.034, FC-log_2_ = −2.4). In the scope of the suspect screening, 15 metabolites (ICL-1: 7; ICL-3: 8) were significantly changed. The most significant altered metabolites were mevalonic acid (ICL = 1, p = 0.032, FC-log_2_ = −1.5), indole (ICL = 1, p = 0.022, FC-log_2_ = 1.2), the fatty acids undecanedioic acid (ICL = 3, score = 0.99, p = 0.001, FC-log_2_ = −1.1), 1,11-undecanedicarboxylic acid (ICL = 3, score = 0.99, p = 0.005, FC-log_2_ = −1.2), and α-aminoadipic acid (ICL = 1, p = 0.027, FC-log_2_ = −1.0), the steroid sulfates cortisol 21-sulfate (ICL = 3, score = 0.96, p = 0.007, FC-log_2_ = −1.1) and estriol 3-sulfate (ICL = 3, score = 0.80, p = 0.023, FC-log_2_ = −1.5), and several metabolites of the heme degradation pathway consisting of biliverdin IXβ (ICL = 1, p = 0.027, FC-log_2_ = −1.4), bilirubin IXα (ICL = 2.a, score = 0.94, p = 0.038, FC-log_2_ = −1.6), and 5-oxo-δ-bilirubin (ICL = 3, score = 0.89, p = 0.013, FC-log_2_ = −1.4). Furthermore, two metabolites of the heme degradation pathway, biliverdin IXα (ICL = 2.a, score = 0.92, p = 0.060, FC-log_2_ = −1.0) and bilirubin IXβ (ICL = 1, p = 0.052, FC-log_2_ = −1.5), were decreased in the pathological group, although not deemed significant based on their p value.

### Neuroactive metabolites are altered in feces of preterm infants with brain injury

The fecal samples were investigated by untargeted metabolomics followed by an evaluation in a purely untargeted fashion combined with a targeted suspect screening for compounds of interest. A positive fold change indicates an increase in the pathological group (Figure 3).

#### Day 7

The untargeted evaluation of the metabolomics data revealed 520 significantly altered features (ICL-1: 2; ICL-2: 6; ICL-3: 91; ICL-4: 145; unidentified: 275) with a log_2_ FC greater than |2|. The most distinct metabolites between experimental groups with high annotation confidence were the bile acids α-phocaecholic acid (ICL = 3, score = 0.88, p = 0.004, FC-log_2_ = 4.0), 1,3,7,12-tetrahydroxycholan-24-oic acid (ICL = 3, score = 0.90, p = 0.016, FC-log_2_ = 2.7), and 3,12-dioxochol-4-en-24-oic acid (ICL = 3, score = 0.75, p = 0.014, FC-log_2_ = 2.4), the neurosteroid epipregnanolone sulfate (ICL = 3, score = 0.99, p = 0.004, FC-log_2_ = 2.7), the tryptophan metabolite indolelactic acid (ICL = 2.a, score = 0.87, p = 0.048, FC-log_2_ = 4.9), 2-hydroxy-3-methylbutyric acid (ICL = 3, score = 0.92, p = 0.016, FC-log_2_ = 2.3), 2-hydroxy-3-(sulfooxy)benzoic acid (ICL = 3, score = 0.75, p = 0.017, FC-log_2_ = 6.8), glucuronic acid (ICL = 3, score = 0.95, p = 0.004, FC-log_2_ = 3.0), and the dipeptides valylproline (ICL = 3, score = 0.99, p = 0.008, FC-log_2_ = −2.3) and valylvaline (ICL = 3, score = 0.83, p = 0.016, FC-log_2_ = −2.4), whereas epipregnanolone sulfate and indolelactic acid were also part of the suspect screening. In the scope of the suspect screening, five metabolites (ICL-1: 2; ICL-2: 1; ICL-3: 2) were significantly altered between groups, including uracil (ICL = 1, p = 0.041, FC-log_2_ = 3.4), methionine sulfone (ICL = 1, p = 0.004, FC-log_2_ = −4.6), and the steroid sulfate corticosterone sulfate (ICL = 3, score = 0.86, FC-log_2_ = 1.4, p = 0.048). Another tryptophan metabolite, indoxyl sulfate (ICL = 3, score = 0.83), was found on day 7 in the majority of samples from the pathological group but was not observed in the control group. In addition, the levels of the neurotransmitter histamine (ICL = 3, score = 0.96, p = 0.368, FC-log_2_ = 6.9), the neuroactive tryptophan metabolite tryptamine (ICL = 1, p = 0.269, FC-log_2_ = 3.3), and the steroid sulfates 16α-hydroxydehydroepiandrosterone 3-sulfate (ICL = 3, score = 0.76, p = 0.073, FC-log_2_ = 1.2) and pregnenolone sulfate (ICL = 3, score = 0.81, p = 0.109, FC-log_2_ = 1.8) were all elevated in the pathological group, although not significant based on their p values.

#### Day 28

Overall, 51 significantly altered features (ICL-2: 1; ICL-3: 15; ICL-4: 7; unidentified: 28) with a log_2_ FC greater than |2| were observed during the untargeted evaluation. The most significantly altered metabolites with high annotation confidence were the bile acid 7β-hydroxy-3,12-dioxo-5β-cholanic acid (ICL = 3, score = 0.93, p = 0.016, FC-log_2_ = −2.7), the tryptophan metabolite 5-hydroxyindole (ICL = 3, score = 0.94, p = 0.005, FC-log_2_ = −2.7), the dopamine metabolite 5-hydroxyindoleacetic acid (ICL = 3, score = 0.90, p = 0.035, FC-log_2_ = −2.5), glucuronolactone (ICL = 3, score = 0.97, p = 0.001, FC-log_2_ = −5.7), and the dipeptide prolylhydroxyproline (ICL = 3, score = 0.86, p = 0.020, FC-log_2_ = −3.3). During the suspect screening, only dopamine (ICL = 1, p = 0.042, FC-log_2_ = −0.8) and adipic acid (ICL = 3, score = 0.76, p = 0.004, FC-log_2_ = 1.7) were deemed significantly changed.

#### Term-equivalent age

Overall, nine features (ICL-3: 6; ICL-4: 2; unidentified: 1) with a log_2_ FC greater than |2| were significantly altered during untargeted evaluation, and no significantly changed metabolites were found during the suspect screening. The most distinct metabolites between experimental groups were nicotinic acid (ICL = 3, score = 0.96, p = 0.010, FC-log_2_ = −2.1) and valyl-prolyl-valine (ICL = 3, score = 0.79, p = 0.003, FC-log_2_ = 2.2).

### Correlations between gut microbiome and neuroactive compounds observed in feces

**Figure 4:**
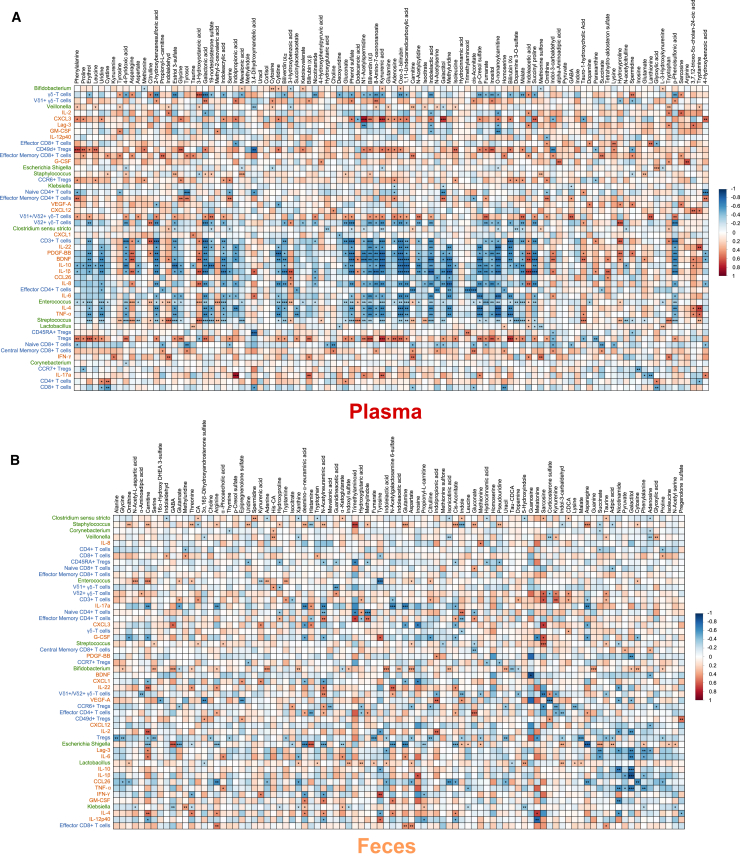
Multi-omics correlation matrix Correlation matrix of 16S rRNA microbiome, T cell, cytokine, and growth factor data with metabolomics data over all time points of (A) plasma (T cell: n = 27; cytokines and growth factors: n = 23; 16S rRNA: n = 78) and (B) feces (T cell: n = 34; cytokines and growth factors: n = 27; 16S rRNA: n = 71). ∗p < 0.05, ∗∗p < 0.01, ∗∗∗p < 0.001.

The results of the 16S rRNA gene amplicon sequencing of the fecal samples, and the cytokine, growth factor, and T cell profiles obtained from this cohort of extremely premature infants in our previous study^2^ were correlated with the data from the targeted and untargeted metabolomics experiments (Figure 4) over all sampling time points and samples available for this study.

#### Plasma

In plasma (Figure 4A), tryptamine was significantly positively correlated with pro-inflammatory factors interleukin-22 (IL-22), tumor necrosis factor α (TNF-α), and CXCL12, which play a role in neurogenesis and neuroinflammation.^27^ Other TRP metabolites, namely indolepropionic acid (ICL = 1), indoleacrylic acid, and indolelactic acid, were negatively correlated with *Enterococcus* and *Streptococcus* and positively with the growth factors brain-derived neurotrophic factor (BDNF) and platelet-derived growth factor BB (PDGF-BB) as well as CXCL3, a chemokine associated with neuroprotective and anti-inflammatory activities,^28^ while for indoleacetic acid and indol-3-carbaldehyde (ICL = 1) the exact opposite trend was visible. Bilirubin, biliverdin, oxo-δ-bilirubin, and the compounds *p*-cresol sulfate, kynurenic acid, and galactonic acid were all negatively correlated with the pro-inflammatory factors γδ-T cells, Vδ2^+^ γδ-T cells, TNF-α, IL-6, IL-1β, and IL-8, while at the same time they were positively correlated with the anti-inflammatory factors regulatory T cells and CXCL3. *Enterococcus*, *Streptococcus*, and γδ-T cells were positively correlated with 3-hydroxyoctanoic acid (ICL = 3, score = 0.78) as were the growth factors BDNF and PDGF-BB. For the compounds phenylalanine, tyrosine, serine, adenosine, galactitol, methionine sulfone, and 4-hydroxybenzoic acid (ICL = 3, score = 0.79), a negative correlation with naive CD4^+^ T cells and effector memory CD4^+^ T cells was observable. *Klebsiella* exhibited negative correlations with galactitol and GABA. Further correlations are visualized in Figure 4A.

#### Feces

We observed significant negative correlations between *Klebsiella* and hydroxyproline (ICL = 1), phenylalanine (ICL = 1), and 5-hydroxyindole (Figure 4B). Significant negative correlations were also observed with the neurotransmitter GABA (ICL = 1) and *N*-acetyl-L-aspartic acid (ICL = 1), the precursor of the neuronal dipeptide *N*-acetylaspartylglutamate. *Staphylococcus* exhibited strong positive correlations with trimethylaminoxide (ICL = 1), glutamine, asparagine, and the sialic acid *N*-acetylneuraminic acid (ICL = 3, score = 0.84), but was negatively correlated with the neuroactive TRP metabolite indole. Tryptamine, also a neuroactive TRP metabolite, was positively correlated with *Enterococcus*, as were carnitine (ICL = 1) and *N*-acetyl-L-aspartic acid (ICL = 1). There was a strong negative correlation between *Enterococcus* and the abundance of the amino acid tyrosine (ICL = 1) in the feces. *Bifidobacterium* showed significant positive correlations with the TRP metabolites indolelactic acid and indoleacetic acid (ICL = 1), GABA, and the nucleobases cytosine (ICL = 1), uracil, and adenine (ICL = 1). Negative correlations were found for the taurine conjugate CDCA (ICL = 1). The strongest correlations between metabolites and the 16S rRNA gene amplicon data were observed in *Escherichia-Shigella*, consisting of strong negative correlations with the amino acids asparagine, arginine, glutamate, and glutamine. *Escherichia-Shigella* also exhibited significant positive correlations with GABA and the TRP metabolites indole-3-carbaldehyde (ICL = 1) and indole. The sialic acids and intestinal mucus components deamino-neuraminic acid (ICL = 3, score = 0.75), *N*-acetylneuraminic acid, and *N*-acetylgalactosamine 6-sulfate (ICL = 3, score = 0.76) were all significantly negatively correlated with IL-17a and *Escherichia-Shigella* while being positively correlated with IL-22. Further correlations are depicted in Figure 4B.

### Bile acid profiles of plasma and feces in extremely premature infants

We batch-synthesized more than 120 reference standards including the majority of thus far described bacterial-derived conjugates of bile acids and amino acids,^29^ consisting of the primary bile acids CA and CDCA, the secondary bile acids UDCA, DCA, HDCA, and LCA, 19 amino acids, and taurine. A targeted LC-MS/MS method for the free bile acids and their respective conjugates was developed, allowing the quantification of >120 analytes while ensuring the chromatographic separation of the isomeric bile acids CDCA, HDCA, DCA, and UDCA and their respective conjugates.

#### Plasma

**Figure 5:**
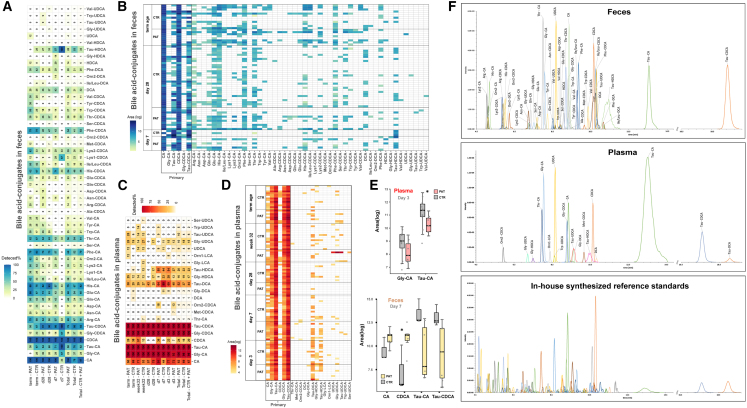
Profiles of bile acids and their conjugates in feces and plasma (A and C) Presence of bile acids (%) in experimental groups at different time points. (B and D) Bile acid profiles of individual samples. (E) Box plots of bile acids altered between experimental groups (plasma: n_CTR_ = 14, n_PAT_ = 10; feces: n_CTR_ = 3, n_PAT_ = 8). ∗p < 0.05, ∗∗p < 0.01, ∗∗∗p < 0.001. (F) Chromatographic peaks of the detected analytes.

Six of the recently described bile acid conjugates were detected (Figure 5D) in the plasma samples, interestingly including ornithine-LCA, which was not detected a single time in its free form or conjugated form in the fecal samples. Furthermore, taurine conjugates of the secondary bile acids HDCA and DCA were already observed in most samples at day 3. In addition, lower levels of taurine-CA (Tau-CA) (p = 0.036, FC-log_2_ = −1.5) and glycine-CA (Gly-CA) (p = 0.110, FC-log_2_ = −1.1) in the pathological group were detected at day 3.

#### Feces

In the fecal samples of extremely premature infants, we identified 40 of the recently discovered bile acid conjugates of five bile acids (Figure 5B), with the exception of lithocholic acid conjugates. A clear trend between the abundance of the primary bile acids CDCA (p = 0.033, FC-log_2_ = 3.3), CA (p = 0.110, FC-log_2_ = 1.6), and their taurine conjugates (Tau-CDCA: p = 0.067, FC-log_2_ = −3.0; Tau-CA: p = 0.110, FC-log_2_ = −3.0) between the experimental groups was observed (Figure 5E), although only one was regarded as significant based on their p values.

## Discussion

### Time-dependent development is the dominating factor in overall differences in plasma and fecal metabolome

Generally, the metabolome of the pathological and control groups exhibited no group-specific clustering in the PCA. However, there was clear clustering between sampling time points, as seen in plasma and feces (Figures 1B and 1C). This finding was supported by the PERMANOVA, which found that time point was a significant factor while group affiliation was not deemed to be significant. This indicates that metabolic changes specific for the differentiation between the pathological and control groups might be only based on a subset of metabolites, and in general individual factors dominate the differences in the metabolome between patients. The plasma metabolome in the early time points, especially on day 7, displayed a less tight clustering and seemed to exhibit more spread compared to term-equivalent age (Figure 1C), indicating a higher susceptibility to variation during earlier periods of infant development. At term-equivalent age a tight clustering between samples was seen, indicating general stabilization and convergence of the plasma metabolome over time. The opposite trend was observed in the fecal samples (Figure 1B), where a slightly tighter clustering was shown at day 28 accompanied by a trend to spread out at term-equivalent age, indicating a diversification of the gut metabolome over time and possibly also the gut microbiome.^30^

### Metabolic differentiation between experimental groups is detectable in the earliest sample time point in both plasma and feces

Despite the findings above, significant changes in several metabolic pathways between the pathological and control groups were already observed at the earliest time points (Figures 2A and 2B). Pathways related to the metabolism of several amino acids dominated in plasma from day 3 to week 32 while being not affected at the last time point. Carbohydrate metabolism was most prominent in feces at day 7, while at term-equivalent age several pathways related to fatty acids and amino acid metabolism were the driving force. At day 28 (feces), a mix of altered carbohydrate and amino acid metabolism was observed, closing the gap between the previous and following time point. In general, a development over time is apparent between different sampling time points in both plasma and feces, where the affected pathways during term-equivalent age are most distinct from the other time points. Interestingly, time point day 28 in feces and plasma exhibited the most significantly changed pathways, in both cases including sialic acid metabolism. This pathway includes *N*-acetylneuraminic acid, which participates in the brain as an integral part of ganglioside structure in synaptogenesis.^31^ Furthermore, in feces, at all three time points a significant or near-significant enrichment was found in biopterin metabolism. Being a cofactor for aromatic amino acid hydroxylases, biopterin is involved in the synthesis of neurotransmitters including dopamine and serotonin.^32^ A difference in the presence and abundance of several metabolites in both plasma and feces was already observed between experimental groups in the first week of life, many of them associated with neuroactivity, neuroprotection, neurotoxicity, and immunomodulatory activity. In Figures 3A–3G, a portion of these differentiated metabolites are highlighted. Several amino acids were significantly increased in plasma samples from the pathological group at day 3, mainly alanine, aspartic acid, serine, glycine, proline, lysine, homoserine, citrulline, and glutamine, which is the principal excitatory neurotransmitter in the brain.^33^ One interesting compound elevated in the pathological group at day 3 was galactitol, a reduction product of galactose. Elevated levels are associated with the presence of galactosemia^34^ and other genetic diseases marked by the deficiency in galactitol metabolism. In addition, it has been implicated for its harmful neurotoxic properties at elevated concentrations and is connected to brain damage^35^^,^^36^ and retinopathy.^34^^,^^37^ Sorbitol, a reduction product of glucose, was associated with peripheral neuropathy^38^ as well as retinopathy,^37^ like galactitol. Mannitol, a commonly used additive in medication, elevates blood plasma osmolality, thereby reducing cerebral edema, elevated intracranial pressure, and cerebrospinal fluid volume and pressure.^39^ The potential of these two compounds to cause retinopathy is especially interesting, since all infants in the pathological groups developed ROP and furthermore was significantly associated with the pathological group. Comparing the galactitol levels at day 3 between infants with and without ROP regardless of experimental group revealed a significant association between increased galactitol levels at day 3 and the development of ROP (Wilcoxon rank-sum test: p = 0.013). Although a reference standard for galactitol was acquired (which confirms the identity at an ICL 1, Figure S2B),^40^ it cannot be excluded that it is its stereoisomer mannitol/sorbitol or a mixture of them, since their retention time and MS/MS spectra might be identical. Interestingly, at the same time a decrease in the alternative galactose metabolite galactonic acid was observed (Figure 3B), indicating altered galactose metabolism between experimental groups shortly after birth. Another elevated neurotoxic compound was *p*-cresol sulfate at day 28 (although not deemed significant, p = 0.067), the sulfate conjugate of the bacteria-derived compound *p*-cresol.^41^ It is associated with neurological impairments in relation to kidney disease, disease progression in Parkinson’s disease,^42^^,^^43^ and neurotoxicity.^44^ A decrease of lanthionine, a biomarker for H_2_S production, was observed in plasma at day 7, indicating that there might be a difference in hydrogen sulfide-related metabolism in the first week after birth. A strong increased FC-log_2_ of histamine was also observed at day 7 in the feces, although not regarded as significant. Histamine is an important neurotransmitter, especially in relation to immune cells, that influences various brain functions such as control of pituitary hormone secretion and cognitive functions^45^ and is known to be secreted by the human gut microbiome. Increased levels of lactate and ratio of lactate/pyruvate at birth have been used as a predictor of occurrence of neonatal encephalopathy in term and preterm infants in another study.^46^ We found a similar distribution of the lactate/pyruvate ratio (Figure S6) for brain injury in the pathological group, although it was not statistically significant.

### Altered tryptophan metabolites and their activity with the CNS

Several compounds related to TRP metabolism with relevant biological activity, some likely of bacterial origin, were found to be significantly altered between experimental groups (Figure 3A). Tryptamine was increased in plasma at day 3 in the pathological group while being decreased at day 7. In feces, increased levels were found at day 7, although not deemed significant. Tryptamine is a bacterial neuroactive TRP metabolite generated by multiple bacterial taxa^10^ and has been associated with neurotoxicity, neurodegeneration, and the onset of autism spectrum disorder (ASD) and schizophrenia.^8^^,^^47^^,^^48^^,^^49^^,^^50^ It modulates intestinal homeostasis by acting on the enteric nervous system and can induce the release of serotonin and ion secretion by intestinal epithelial cells.^51^ Indolelactic acid, a metabolite produced by *Bifidobacterium*,^52^ was significantly increased in the pathological group at day 7 in both plasma and feces. This TRP metabolite was found to impact immune function in early life^53^^,^^54^ and to be elevated in children with ASD.^55^ Moreover, it also has been suggested to exhibit neuroprotective properties in infants,^53^ and its levels were found to strongly correlate with inflammation responses in humans.^56^ Another TRP metabolite affected in plasma and decreased at day 7 was indoleacrylic acid, which promotes intestinal epithelial barrier function and mitigation of inflammatory responses, thereby having an important anti-inflammatory function in the intestine.^57^ 5-Hydroxyindole was found to be significantly decreased in the pathological group in feces at day 28 and is known to stimulate gastrointestinal motility^58^ and affect the α-7-nicotinic receptor,^59^^,^^60^^,^^61^ which has a potential role in the reduction of inflammatory and neurotoxicity in sepsis, myocardial infarction, stroke, and Alzheimer’s disease.^62^^,^^63^^,^^64^ Indole was also increased in plasma of the pathological group at term-equivalent age and has been found at high levels in children with ASD.^8^ Indoxyl sulfate, an indole metabolite, was increased in the pathological group at day 7 in the feces. It has been associated with ASD in children^55^ and acts as an agonist of the aryl-hydrogen receptor (AhR),^65^ a receptor in the gut-brain axis^66^ that is involved in inflammatory and neoplastic diseases.^67^ Kynurenic acid, a metabolite of the kynurenine pathway of endogenous TRP metabolism, was significantly decreased in plasma at week 32 (Figure 3G). It is known to interact with AhR, glutamate, and *N*-methyl-D-aspartic acid (NMDA) receptors and has influence on important neurophysiological and neuropathological processes including neuroprotection.^68^^,^^69^ Furthermore, kynurenic acid is also involved in the immune system and inflammation, exhibiting anti-inflammatory and immunosuppressive functions.^70^

### Steroid sulfates and their role in the gut-brain axis and neurodevelopment

At day 3 and week 32 in plasma, dopamine 3-*O*-sulfate was significantly decreased in the pathological group. In human blood circulation endogenous dopamine exists predominantly in the sulfated form, and sulfonation is an important regulation process, since the sulfated form does not bind to dopamine receptors.^71^ Furthermore, dopamine itself and its metabolite 5-hydroxyindoleacetic acid were significantly decreased in the feces of the pathological group at day 28. Both in feces and plasma, the abundance of several steroid sulfates was altered between experimental groups at different time points (Figures 3D and 3E). Steroids play a role in neuromodulation and are contributors to gut-brain-axis signaling. Furthermore, they are influenced by the gut microbiome, presumably through modulating GABA_A_ and NMDA receptors.^72^ Steroid sulfation and desulfation greatly affect the biological activity, regulating the steroid bioactivity and, in many cases, unconjugated and sulfated steroids acting in opposite ways on the same receptors. Steroid sulfates also have an important role in the regulation of steroid metabolism and transport.^73^ Sulfation also increases the polarity, thereby reducing their passage through the blood-brain barrier (BBB). Furthermore, steroid sulfonation by gut bacteria has been associated with alteration of immune cell trafficking in the host.^74^ A higher abundance of neurosteroids, including epipregnenolone sulfate and pregnenolone sulfate (not deemed significant), were found in the feces of the pathological group at day 7. Epipregnenolone sulfate and pregnenolone sulfate are neurosteroids and agonists of TRPM3^75^ and negative allosteric modulators of GABA_A_^76^ and NMDA receptors,^77^ both being relevant parts of the gut-brain axis.^78^^,^^79^ Furthermore, hypofunction of NMDA receptors was associated with impairment of synaptic plasticity.^80^ In plasma a decrease in several steroid sulfates was observed at day 3 and then again at week 32 and term-equivalent age, including estriol 3-sulfate. Estriol can only be produced from fetal precursors^81^ and plays a particularly prominent role in the context of pregnancy and fetal development. It also exhibited neuroprotective properties in multiple sclerosis by reversing myelin breakdown in the brain. A clear interpretation of altered steroid sulfate levels is difficult, since there are several potential causes such as a change in sulfonation or desulfonation, increased excretion, and augmented uptake into tissue. Mevalonic acid, which was decreased in plasma at term-equivalent age (Figure 3G), is essential for inducing trained innate immunity and is an integral part of cholesterol synthesis.^82^ Besides being the precursor of the synthesis of other steroids, cholesterol is also an important constituent of neuronal myelin and is crucial for brain development during the embryonic and postnatal stages.^83^

### Brain- and immune-system-affecting plasma metabolites differentiate at later time points

During enrichment analysis (Figure 2B), the metabolic pathway porphyrin metabolism, including heme degradation, was significantly altered between experimental groups at term-equivalent age in plasma. Abundance of the heme degradation metabolites biliverdin, bilirubin, and 5-oxo-δ-bilirubin were significantly decreased in the pathological group at term-equivalent age (Figure 3F). Biliverdin and 5-oxo-δ-bilirubin are products of heme catabolism, and biliverdin is further metabolized to bilirubin, which has been associated with neurological dysfunction and neurotoxicity.^84^^,^^85^ Increased levels of bilirubin lead to jaundice, and in infants the underdeveloped BBB of neonates allows bilirubin to diffuse across and accumulate in the brain, causing permanent brain damage. Besides these negative effects, bilirubin and biliverdin have been linked to numerous anti-inflammatory and immunosuppressive actions and regulation of the innate immune system.^86^^,^^87^ Furthermore, they are known activate the AhR.^88^ The decrease of bilirubin and biliverdin might be caused by an altered heme abundance or heme degradation. Another possibility is increased excretion, although no difference in their abundance or of their metabolites was observed in the feces. An increased permeation through an underdeveloped BBB may also occur, potentially contributing to brain injury owing to the neurotoxic effects of these compounds. We observed several significant correlations between the metabolomics data and the 16S rRNA gene amplicon, cytokine, growth factor, and T cell data. Plasma levels of heme degradation metabolites were decreased in the pathological group at term-equivalent age, and positive correlations with anti-inflammatory and neuroprotective factors were observed while correlations with pro-inflammatory factors were negative (Figure 4A). The same trend was observed for galactonic acid and indoleacrylic acid. A significant negative correlation between galactitol in plasma and *Klebsiella* was observed. This aligns well with increased levels of the neurotoxic galactitol in plasma at day 3 in the pathological group and the decreased levels of *Klebsiella* in the first week of life.^30^ *Klebsiella* overgrowth at day 28 was previously associated with brain injury in extremely premature infants and could be a potential predictor of pathological neurophysiological development. The same trend was found in the feces, where there was a negative correlation between *Klebsiella* and the TRP metabolite 5-hydroxyindole, both significantly altered at day 28. Several steroid sulfates, found to be differently abundant between experimental groups in the feces, were significantly negatively correlated with vascular endothelial growth factor A (VEGF-A), which was associated with pathological brain development in premature infants.^2^

### Differentiation of bile acids and potential differences in metabolic activity of the gut microbiome

Recently, more than 145 as yet undescribed bacterial-derived bile acid amino acid conjugates^29^ were discovered that have been connected to disease phenotypes in Crohn’s disease, ulcerative colitis, and disease severity in inflammatory bowel disease.^89^ Initiated by this recent discovery, we batch-synthesized more than 120 bile acid conjugates consisting of CA, CDCA, DCA, HDCA, UDCA, and LCA coupled to 19 amino acids and taurine, followed by the development of a targeted LC-MS/MS method for detection and quantitation of those bile acids and their conjugates. Utilizing this developed assay, we detected 40 of the recently discovered bile acid conjugates in the fecal samples (Figures 4A and 4B), consisting mainly of primary bile acid amino acid conjugates of CA and CDCA, although secondary bile acid amino acid conjugates of DCA, HDCA and UDCA were also detected. In the fecal sample at day 7 (Figure 5E), a significantly higher abundance of CDCA was observed for the pathological group, while for the taurine-conjugated form of CDCA an opposite trend was observed. In the plasma, no significant changes for CDCA or Tau-CDCA at were found at day 7. Chenodeoxycholic acid is conjugated to taurine in the liver, and the resulting Tau-CDCA is excreted via the bile into the intestinal tract where it can be reabsorbed into the bloodstream (i.e., enterohepatic circulation). It can also be deconjugated back to CDCA and metabolized to secondary bile acids or other bile acid metabolites by the gut microbiome. This increase in CDCA coupled with a decrease in Tau-CDCA in feces might be an indicator of a general or a bile acid specific elevation of bacterial metabolism in the intestinal tract. CDCA has been shown to decrease *trans*-epithelial electrical resistance, increase intestinal permeability, and elevate levels of IL-8, IL-6, TNF-α, and VEGF release.^90^ In contrast to the fecal samples, only six of the bile acid amino acid conjugates were sparingly detected in plasma samples (Figures 5C and 5D). It is not clear whether this was caused by the absence of those compounds or by the expected lower concentration in the blood compared to feces, rendering them to be under the limit of detection. The taurine and glycine conjugates of the gut-microbiome-derived secondary bile acids HDCA, DCA, and, partially, UDCA were already detected in plasma samples at day 3. This observation might indicate that 3 days after delivery there is already a metabolically active gut microbiome established, producing several bile acid transformation products which are reabsorbed into the bloodstream with the potential of affecting the host. The findings in our previous publication^2^ showed the presence of a gut microbiome in fecal samples as early as day 3, exhibiting already significant differences between the experimental groups. However, it is also known that primary and secondary bile acids in their free form can be transferred through breast milk from the mother to the infant,^91^^,^^92^ and to a much lesser degree glycine and taurine conjugates.^93^ Therefore, it cannot be excluded that these secondary bile acids might originate partially or altogether from the consumed breast milk. At day 3, lower plasma levels of the primary bile acid conjugates Tau-CA and Gly-CA were observed in the pathological group (Figure 5E). The reported changes in bile acids at the earliest sampling time points in both plasma and feces clearly strengthen the observations from the untargeted metabolomics experiments, observing the same early onset of differentiation in metabolites between experimental groups. In general, there was no clear distinction in the occurrence of the amino acid conjugates between experimental groups, with their presence seeming to be a highly individual factor (Figure 5A).

### Limitations of the study

Due to the nature and circumstances of extremely premature infants, it is exceptionally difficult to obtain samples in greater number from the same cohort or same patient. This results in typically smaller sample size but also a limited amount of possible sampling time points with greater time gaps in between. Furthermore, this makes it difficult to obtain a fully longitudinal sample set. Also, the sample amount available is limited, making the sample preparation and subsequent measurements more challenging. Another problematic factor is the potential for different onset times in the pathological processes behind brain injury among patients. This potentially delayed onset combined with the greater time gaps between sampling time points poses a major problem for a quantitative time-course investigation, especially regarding statistical analysis, since not every patient might be in the exact same step of pathological progression at the sampling time point. At the early time points this can lead to missing values of potential key metabolites, and combined with the small sample size this increases the difficulty of analysis and reduces the statistical power needed for deeming changes as significant. In addition, for one sampling time point (feces day 7) only a very low number of samples in the control group was available; therefore, the results regarding this group must be interpreted with caution. Furthermore, the annotation of some significantly altered metabolites are based on *in silico* tools, so a false-positive identification cannot be excluded. In general, it is challenging to differentiate between potential causes and symptoms of pathological neurological development due to metabolic and bacterial changes. An early neurological injury might cause differences in various areas such as feeding, antibiotic use, gastrointestinal motility, or development of the BBB, causing metabolic or microbial changes later on. The observed correlations between 16S rRNA gene amplicon data and metabolites warrant future studies employing metagenomic analyses to investigate the metabolic potential of the observed bacterial strains and draw a stronger conclusion regarding the relation between the observed metabolites and the microbiome. Despite these difficulties, we were able to collect the most comprehensive sample set to date over several time points of extremely premature infants and obtained comprehensive and highly relevant insights by employing highly optimized sample preparation procedures and MS workflows to deal with the very low sample amounts available.

### Conclusion and outlook

The occurrence of extremely premature births is rising globally, leading to an ever-growing highly vulnerable patient group. Although survival rates of extremely premature infants have been increasing as a result of improved neonatal intensive care, a considerable number of infants suffer from severe morbidity and life-long neurodevelopmental impairment. In this work we determined the metabolic profile of plasma and fecal samples from different sampling time points in two groups of patients based on MRI findings at term-equivalent age. Despite the limited sample amount and the difficult nature of the biological background of this patient group, we observed relevant molecular markers, indicating an early onset of differentiation between the group with brain abnormalities and the control group without any abnormalities. This distinction is characterized by a difference regarding the abundance and presence of primary bile acids/conjugates and numerous other compounds with relevant bioactivities, some of them derived from the gut microbiome. This includes neuroactivity, neurotoxicity, neuroprotection, immunomodulation, and inflammatory and anti-inflammatory actions. Furthermore, a broad array of significantly altered molecules is known to interact with AhR, GABA, and NMDA receptors, which are part of the gut-brain axis and are involved in the immune system, CNS, inflammation, neurodevelopment, and synaptic plasticity. Several compounds correlated to pro- and anti-inflammatory factors were significantly altered between the pathological group and the control group. These compounds might be beneficial in early diagnosis of pathological development. Furthermore, microbially derived secondary bile acids and bile acid amino acid conjugates were already present in the plasma 3 days after delivery, suggesting the establishment of an already metabolic active gut microbiome and the absorption of its metabolites into the bloodstream. This would indicate that the potential early influence of the gut microbiome on its host is not limited to the gut-brain axis but already takes place via direct uptake of metabolites. The findings in this study help to better understand the cause and development of brain impairments in extremely premature infants and their connection to the gut microbiome and lay the foundation for mechanistic studies.

## STAR★Methods

### Key resources table

**Table:** 

REAGENT or RESOURCE	SOURCE	IDENTIFIER
**Biological samples**		

Peripheral blood samples from extremely premature infants	General Hospital of Vienna	N/A
Fecal samples from extremely premature infants	General Hospital of Vienna	N/A

**Deposited data**		

Raw metabolomics data	https://www.ebi.ac.uk/metabolights/editor/MTBLS7560	Metabolights: MTBLS7560
Supplementary spectral data	https://phaidra.univie.ac.at/o:2044701	Phaidra: 2044701
16S rRNA gene amplicon sequencing, T-cells, cytokines, growth factor data	https://doi.org/10.1016/j.chom.2021.08.004	NCBI: PRJNA715072

**Chemicals**, **peptides**, **and recombinant proteins**		

Water LC-MS grade	VWR	Cat # 83645.320P
Acetonitrile LC-MS grade	Honeywell Riedel de-Haën	Cat # 14261
Methanol LC-MS grade	Honeywell Riedel de-Haën	Cat # 14262
Formic acid	Promochem	Cat # FLK.14265.0050.GF
In-house chemical reference standard library	METHOD DETAILS/Chemicals and reagents	Table S1
SRM 1950^107^	NIST, Gaithersburg, US	https://doi.org/10.1021/ac402503m

**Software and algorithms**		

Python (3.8)	Python Software Foundation	https://www.python.org
R (4.1)	The R Project for Statistical Computing	https://www.R-project.org/
XCMS (3.14)	Smith et al.^94^	https://bioconductor.org/packages/release/bioc/html/xcms.html
Proteo Wizard (3.0.18)	Chambers^95^	https://proteowizard.sourceforge.io/
CAMERA (1.48)	Kuhl et al.^96^	https://www.bioconductor.org/packages/release/bioc/html/CAMERA.html
NeatMS (0.9)	Gloaguen et al.^97^	https://github.com/bihealth/NeatMS
SIRIUS 4 (4.9)	Dührkop et al.^98^	https://bio.informatik.uni-jena.de/software/sirius-4-0-1/
Skyline (20.2)	Adams et al.^99^	https://skyline.ms/project/home/software/Skyline/begin.view
qvalue (2.28)	Storey et al.^100^	https://www.bioconductor.org/packages/release/bioc/html/qvalue.html
GNPS	Aron et al.^101^	https://gnps.ucsd.edu/ProteoSAFe/static/gnps-splash.jsp
MetaboAnalyst (5.0)	Pang et al.^102^	https://www.metaboanalyst.ca/
mummichog (version 2)	Li et al.^26^	https://github.com/shuzhao-li/mummichog
MixOmics (6.20)	Rohart et al.^103^	https://bioconductor.org/packages/release/bioc/html/mixOmics.html
Cytoscape (3.9)	Shannon et al.^103^	https://cytoscape.org/
vegan (2.3)	Oksanen et al.^104^	https://cran.r-project.org/web/packages/vegan/index.html
Hmisc (4.7)	Harrell et al.^105^	https://cran.r-project.org/web/packages/Hmisc/index.html
Nontargeted Analysis Study Reporting Tool	Peter et al.^106^	https://doi.org/10.1021/acs.analchem.1c02621

### Resource availability

#### Lead contact

Further information and requests for resources and reagents should be directed to the lead contact, Benedikt Warth (benedikt.warth@univie.ac.at).

#### Materials availability

This study did not generate new unique reagents.

#### Data and code availability

The raw LC-HRMS and LC-MS/MS data are publicly provided via the Metabolights repository (https://www.ebi.ac.uk/metabolights) under the identifier MTBLS7560. The 16S rRNA gene amplicon sequencing, T-cells, cytokines, growth factor data can be found at NCBI under the identifier PRJNA715072 (https://doi.org/10.1016/ j.chom.2021.08.004). Additional supplementary spectral data can be found under https://phaidra.univie.ac.at/o:2044701. No custom code is reported in this paper. Any additional information required to reanalyze the data reported in this paper is available from the lead contact upon request.

### Experimental model and study participant details

#### Cohort of extremely premature infants and sample collection

This study was approved by the ethics committee of the Medical University of Vienna (Ethics number 1348/2017). In a single cohort of sixty extremely premature infants, neurophysiological development was monitored during hospitalization and brain injuries were identified by a combination of ultrasound imaging at multiple time points and magnetic resonance imaging (MRI) at term-equivalent age. The infants were enrolled between September 2017 and June 2019 at the Medical University of Vienna. All parents gave written-informed consent before study inclusion. Out of the cohort of 60 infants, 53 survived and 38 of the remaining infants displayed age-adequate MRIs or only mild brain injuries while 15 infants were diagnosed with severe pathological brain injuries. Infants diagnosed with age-adequate or only mild brain injuries were assigned to the control group (CTR) while the pathological group (PAT) consisted of infants with severe pathological brain injuries. Among the observed injuries were periventricular hemorrhagic infarctions, intraventricular hemorrhages, cerebellar hemorrhages, and periventricular leukomalacia. A reduction of brain volume and an enlargement of the subarachnoid spaces was accompanying many of those injuries. Out of those surviving infants, samples from 51 infants were available and used for this study (Figure 1A). The distribution of sex assigned at birth within and between experimental groups was close to 50% (CTR: 56% female, PAT: 47% female) and showed no statistical significance (Table 1). Every infant received the probiotic preparation Infloran (*Bifidobacterium bifidum* and *Lactobacillus acidophilus*) from the first day of life until the corrected age of 34 weeks of gestational age. The enteral feeding regimen consisted of the infant’s own pasteurized mother’s milk or pasteurized human donor milk. After achieving 100 mL kg^−1^ enteral feeding, the milk was fortified with 4% bovine milk fortifier until term-equivalent age. Characteristics of the study cohort used in this study can be found in Table 1 and the SI (*SI_Cohort_Information*). Over the course of hospitalization and when possible, fecal samples were collected, and blood was drawn at multiple time points with a focus on day 3, day 7, day 28, correction week 32 and term-equivalent age. The fecal samples from all extremely premature infants were collected from diapers via collection tubes and stored at −80°C until further analysis. For minimizing the stress for patients, blood samples were only taken combined with clinical routine sampling. The blood samples were transferred into Lithium Heparin S-Monovettes and the plasma was separated and stored at −80°C until further analysis. Additional information about the cohort and MRI-results and neurophysiological assessment can be found in previous work.^2^

### Method details

#### Chemicals and reagents

Acetonitrile (ACN), methanol (MeOH) and water were LC-MS grade and purchased from VWR Chemicals (water) or Honeywell Riedel de-Haën (ACN, MeOH). Formic acid (FA) used as an additive for eluents was purchased from Promochem. Chemical reference standards used during LC-MS measurements were acquired from Sigma Aldrich, Carbosynth or Toronto Research Chemicals. The bile acid conjugate reference standards were synthesized as described in the supporting information (Figure S1A), and the used reagents were purchased from Sigma Aldrich. Isotopically labeled compounds used as internal standards, were purchased from Cambridge Isotope Laboratories.

#### Batch synthesis of bile acid conjugates

A solution of a single bile acid (40 μL, 2.5 mM, 1 eq) was combined with freshly prepared solutions of triphenylphosphine (40 μL, 10 mM, 4 eq) and dipyridyldisulfide (40 μL, 10 mM, 4 eq), vortexed and incubated for 1 min. A amino acid stock solution (5 μL, 2 mM, 0.25 eq), containing 10 amino acids, was added and the reaction mixture was incubated on a thermo-shaker (TS-100, Biosan) at 60°C for 30 min, followed by the addition of 300 μL of ACN. Taurine conjugates were synthesized in the same manner. This procedure was repeated for all bile acids until all desired compounds were acquired. From each mixture 150 μL were taken, combined, evaporated and reconstituted in 150 μL ACN to generate a concentrated standard.

#### Sample preparation of plasma samples

Plasma samples obtained from premature infants with pathological damage or age-adequate damage were used. The samples were stored at −80°C prior to extraction. During the whole sample preparation, the samples were kept on ice. Homogenization by vortexing was followed by the addition of the extraction solvent (ACN/MeOH, 50:50, v/v) to the homogenized sample, resulting in a 1:4 volume ratio of sample and extraction solvent. After further vortexing and sonication in an ice bath for 15 min, the sample solutions were stored overnight at −20 C°. Afterward the samples were centrifuged at 18,000 x g and 4°C for 10 min and the supernatant was transferred to another tube. For targeted LC-MS/MS analysis of bile acid conjugates, aliquots were taken from the solution and stored at −80°C until further use. The remaining solution was evaporated to dryness at 4°C using a SpeedVac concentrator (Labconco). The residues were reconstituted (ACN/water, 50:50, v/v) to one-fifth of the volume prior to evaporation, vortexed and sonicated in an ice bath for 15 min. After centrifugation at 18,000 x g and 4°C for 10 min, the supernatants were transferred to LC vials and stored at −80°C until analysis. An internal standard (^2^H_4_-ursudeoxycholic acid) was added to the aliquots taken for the targeted LC-MS/MS measurements to a final concentration of 2 μM, followed by a transfer of the samples to LC vials and storage at −80°C until analysis.

#### Sample preparation of fecal samples

Stool samples obtained from premature infants with pathological damage or age adequate damage were used. The samples were stored at −80°C prior to the extraction. During the whole sample preparation the samples were kept on ice. The samples were manually homogenized using a spatula and dried at 4°C using a SpeedVac concentrator (Labconco) for 60 h followed by the addition of MeOH/ACN/H_2_O (40:40:20; v/v:v) at a ratio of 50 μL for each mg of dried sample. After vortexing and sonication on ice for 10 min, the samples were homogenized in a bead shaker (4 m/s, MP FastPrep-24 5G) for 15 s and stored overnight at −20°C. Afterward, the samples were centrifuged at 18000 g and 4°C for 10 min and transferred to another tube. The solution was diluted 1:1 with ACN/H_2_O (20:80; v/v) containing internal standards (^13^C_6_-bisphenol A, ^2^H_4_-genistein). After centrifugation at 18,000 x g and 4°C for 10 min, samples were sonicated in an ice bath for 10 min to dissolve potential precipitate and transferred to HPLC vials and stored at −80°C until analysis.

The fecal samples for the targeted LC-MS/MS analysis of bile acid conjugates were dried as described above, followed by the addition of 25 μL H_2_O for each mg of dried sample and the homogenization in a bead shaker FastPrep-24 5G (4 m/s) for 10 s. Subsequently the samples were centrifuged at 18,000 x g and 4°C for 10 min and half the volume of the supernatant was transferred to another tube and stored for additional biochemical assays. The next step was the addition of MeOH/ACN (1:1; v/v) to a final solution of MeOH/ACN/H_2_O (40:40:20; v/v:v), following the extraction with a bead shaker (4 m/s, MP FastPrep-24 5G) for 10 s. After sonication in an ice bath for 10 min, the sample solutions were stored overnight at −20 C°. Afterward the samples were centrifuged at 18,000 x g and 4°C for 10 min and transferred to another tube. The remaining solution was evaporated to dryness at 4°C in a SpeedVac (Labconco) and the residues were reconstituted with (ACN/H_2_O, 50:50, v/v), containing an internal standard (^2^H_4_-ursudeoxycholic acid, 2 μM), to the same volume before evaporation, vortexed and sonicated in an ice bath for 15 min. After centrifugation at 18,000 x g and 4°C for 10 min, the samples were transferred to HPLC vials and stored at −80°C until analysis.

#### Untargeted metabolomics experiments

For the analysis a Vanquish UHPLC system coupled to a QExactive HF quadrupole-Orbitrap (Thermo) mass spectrometer via an ESI interface was used. Two columns, a HILIC column (SeQuant ZIC-pHILIC; 5 μm, 150 × 2.1 mm) and an RP column (Waters Acquity HSS T3; 1.8 μm, 100 × 2.1 mm) were chosen for complementary and broad coverage of analytes. In both cases a precolumn made of the respective material was used. An injection volume of 3 μL, a flow rate of 0.3 mL/min and a column compartment temperature of 40°C was selected for both columns. The HILIC chromatography utilized H_2_O and ACN with 10 mM NH_4_HCO_3_ (90:10, v/v, pH 9.2, eluent A) and ACN (eluent B) for the following gradient: 0–6 min linear decrease from 75% B to 45%B, 6–7 min 45% B to 30% B, 7–10 min constant at 30% B, 10 min until 10.1 min increase from 30% B to 75% B and maintained constant at 75% B for 5 min. The RP chromatography utilized H_2_O with 0.1% formic acid (eluent A) and MeOH with 0.1% formic acid (eluent B) for the following gradient: 0–0.5 min stay at 5% B, 0.5–11.5 min linear increase to 95% B, 11.5–15.5 hold at 95% B,15.5–15.6 decrease from 95% B to 5% B, 15.6–17 min stay at 5% B. Measurements were conducted in either full scan with polarity switching mode or in positive and negative mode with data dependent MS^2^ collection. The settings of the ESI interface were: sheath gas, 48 au; auxiliary gas, 15 au; sweep gas flow, 2 au; capillary voltage, 3.5 kV (positive), 2.8 kV (negative); capillary temperature, 300°C; auxiliary gas heater, 350°C. The scan range was from *m/z* 62 to 900. For full scan measurements the resolution was set to 60,000 with an automatic gain control (AGC) of 1x10^6^ and a maximum injection time of 200 ms. For generating MS^2^ spectra, data dependent acquisition in combination with an inclusion list with features of interest, based on the untargeted and targeted evaluation of the MS1 data, was used. For the data-dependent MS^2^ acquisition a resolution of 60 000 with an AGC target of 1x10^6^ and a maximum injection time of 100 ms were chosen for the full scan. The settings for the MS^2^ collection were as following: resolution 30,000, AGC target 5x10^5^, maximum injection time 120 ms, loop count 10, isolation window 1.0 *m/z*, stepped collision energy 30–50 eV; minimum AGC 8x10^3^.

#### Targeted LC-MS/MS for bile acids and their conjugates

A targeted LC-MS/MS for the investigation of bile acids and their conjugates was developed employing commercially purchased or self synthesized reference standards (Figure S1). For the targeted LC-MS analysis a Dionex Ultimate 3000 UHPLC (Thermo) system coupled to a TSQ Vantage triple quadrupole mass spectrometer (Thermo) via an ESI interface was used. An Atlantis T3 column (Waters, 3 μm, 3 mm × 150 mm), including a precolumn made of the same material, at a flow rate of 0.6 mL/min was used and maintained at 40°C. The injection volume was 5 μL. Chromatography used H_2_O with 0.1% formic acid (eluent A) and ACN/MeOH (1:1, v/v, eluent B) with 2% H_2_O and 0.1% formic acid for the following gradient: 0–1 min at 5% B; rise to 50% B until 1.5 min; increase to 95% B until 10 min; 10–13 min at 95% B; 13–13.1 min from 95% B to 75% B and maintain until 19 min before re-equilibrating at 5% B until 21 min. Negative ESI mode was used with the following ion source parameters: Capillary temperature of 300 (°C), vaporizer temperature of 300 (°C), sheath gas pressure of 40 (Arb), aux valve flow of 10 (Arb), declustering potential of 14 (V), collision gas pressure of 1.5 (mTorr) and a spray voltage of 3000 (V). The cycle time was set to 0.6 (s), while Q1 and Q3 peak width was kept at 0.7 (FWHM).

#### QC & QA measurements for LC-MS methods

For the untargeted metabolomics measurements multiple measures to assure high quality data were taken. During the sample preparation three process blanks were processed together with the samples to investigate potential contaminations during the procedure and measured together with solvent blanks at the beginning and end of each sequence. For system suitability a mixture of reference standards (2 μM, 159 compounds, Table S1) was measured at the beginning and the end of the sequence, and for monitoring potential carry over between samples, solvent blank injections (ACN/H_2_O, v/v, 1:1) were measured. A pooled QC prepared from all samples was injected at the beginning, the end and after every five samples and analyzed throughout the sequence and were used together with internal standards (^13^C_6_-bisphenol A, ^2^H_4_-genisteine, 2 μM) for monitoring potential analytical deviations during the measurement. An SRM 1950 sample (metabolites in frozen plasma, NIST, Gaithersburg, USA) was extracted according to protocol^107^ and measured at the beginning and the end of a sequence.

During the targeted LC-MS/MS measurements process blanks and solvent blanks were measured at the beginning and end of each sequence. A pooled QC, spiked with the reference standards (n = 126, SI: *SI_MRMS_Targeted_LC_MSMS_Bile_acids*), was injected at the beginning, the end and after every 20 samples, and was used together with an internal standard (^2^H_4_-ursudeoxycholic acid, 2 μM) for monitoring potential analytical deviations during the measurement. The injection order of the samples during LC-HRMS and LC-MS/MS experiments was randomized to avoid any experimental group specific bias. The NTA Study Reporting Tool (SRT)^106^ was used as guidance for the construction of the materials and methods section to ensure high reporting quality.

### Quantification and statistical analysis

#### Statistical analysis of the cohort characteristics

Fisher’s exact test and Student’s t test followed by multiple testing correction with Bonferroni’s method, all performed via R (4.1), were used to analyze the relationship between cohort characteristics and experimental groups (Table 1).

#### Data processing and integration of LC-MS acquired data

Thermo Fisher LC-HRMS(/MS) raw data files from the untargeted metabolomics measurements were converted into mzXML files, centroided and separated into two files according to polarity using Proteo Wizard (3.0.18).^95^ All experimental sampling time points from their respective biological matrix were processed together. Data pre-processing was done using the R (4.1) package XCMS^94^ (3.14), starting with the import of the mzXML files. The next step was peak detection using the *centWave* algorithm (*parameters*: *ppm=5*, *peakwidth=5/15*; *snthresh=10*, *prefilter=3/5000*) followed by the removing of peaks with a too wide peak width. The retention time between samples were aligned employing the *obiwarp* algorithm (*parameters*: *binSize=0*.*6*, *distFun="cor_opt"*) with a previous alignment of pooled QC samples followed by the alignment of the samples. The detected peaks were grouped to features with a minimum threshold of 40% in at least one experimental group (*parameters*: *bw=1*, *minFraction=0*.*4*, *minSamples=5*). Features missing a peak in certain samples were integrated in the respective retention time width of the feature. The resulting feature list was further processed by the R package CAMERA (1.48)^96^ (*parameters*: *perfwhm=0*.*6*, *ppm=5*), creating adduct and isotopologue annotations. Features annotated as isotopologues and features with an adduct annotation different from [M+H]^+^ or [M-H]^-^ were removed from the feature list. In addition, features with a signal in the process blank or solvent blank higher than 20% of the median signal in the pooled QC were excluded. All features eluting in the void volume were also removed from the feature list (HILIC: 1 min, RP: 0.65 min). The Python-based package NeatMS (0.9),^97^ based on neuronal networking, was used to classify the peak quality. Features mainly consisting of the peak label “Noise”/“Low Quality” were filtered out. For each sampling time point a subset of the feature list was created and further processed. Features with more than 20% missing values were removed and the missing values were imputed by using half of the minimum value. Fold changes (log_2_) between groups were determined for each time point, and unknown features found in the untargeted data within a threshold of fold change log_2_ < |2| were considered for further MS2 experiments, annotation and following analysis. Feature annotation was carried out by *in silico* fragmentation employing SIRIUS 4^98^ (*parameter*: *5 ppm*; *possible Ionizations* [*M-H*]*^-^*, [*M*+*Cl*]^-^, [*M*+*H*]^+^, [*M*+*Na*]^+^; *DB*: *Bio database*), based on the received score and manual reviewing, and was expanded by comparing experimental MS^2^ spectra with spectral libraries using GNPS^101^ (*parameters*: *Score Threshold*: *0*.*7*, *Precursor Ion Mass Tolerance*: *0*.*05*, *Min Matched Peaks*: *5*) whenever possible. Features annotated by SIRIUS employing fragmentation based molecular formula prediction were assigned an identification confidence level (ICL) of 4, features annotated by *in silico* fragmentation an ICL of 3, features with an unambiguous match in a spectral library with an ICL of 2.a, while compounds with an identity confirmed by reference standards, were assigned an ICL of 1.^40^ In addition to the untargeted evaluation, a targeted suspect screening of compounds (Table S2) with available reference standard and/or compounds of high interest based on previous measurements was done using the software Skyline (20.2).^99^ Fold changes (log_2_) between groups were determined and corresponding p values were calculated using Wilcoxon Rank-sum test. The raw files obtained from the LC-MS/MS experiments targeting bile acids and their conjugates were also processed and quantified by Skyline (20.2). Fold changes (log_2_) between groups were determined and corresponding p values were calculated using Wilcoxon Rank-sum test. FDR was controlled using the ‘qvalue’ (2.28)^100^^,^^108^ R package with a cutoff of <0.2. For a putative Gene Set Enrichment Analysis (GSEA) and over-representation analysis (ORA, mummichog^26^) the ‘MS Peaks to Pathways’ tool from the web application MetaboAnalyst (5.0)^102^ was used (*parameters*: p *value cutoff*: *0*.*1*, *mummichog*+*GSEA*, *min*. *5 entries*, *MFN DB*). For this purpose, the datasets of the HILIC and the RP chromatography and positive and negative polarity mode were combined to one dataset for each time point. The activity network was created using the Python (3.8) package mummichog (version 2)^26^ and was visualized using Cytoscape (3.9).^109^ Multivariate statistical analysis (PCA) was done using the R package ‘MixOmics’ (6.20)^103^ on the auto-scaled and combined data of both positive and negative polarity of RP and HILIC chromatography. Correlation analysis was done using the R package ‘Hmisc’ (4.7)^105^ based on Spearman’s correlation and for the PERMANOVA and dispersion analysis the R package ‘vegan’ (2.3)^104^ was used.

## Supplemental information

Document S1. Figures S1–S5

Table S1. Detailed information about the cohort of extremely premature infants, related to “cohort of extremely premature infants and sample collection” in STAR Methods

Table S2. Additional data regarding the metabolomics experiments of feces samples from sampling time point day 7, related to “data processing and integration of LC-MS acquired data” in STAR Methods

Table S3. Additional data regarding the metabolomics experiments of feces samples from sampling time point day 28, related to “data processing and integration of LC-MS acquired data” in STAR Methods

Table S4. Additional data regarding the metabolomics experiments of feces samples from sampling time point term-equivalent age, related to “data processing and integration of LC-MS acquired data” in STAR Methods

Table S5. Additional data regarding the metabolomics experiments of plasma samples from sampling time point day 3, related to “data processing and integration of LC-MS acquired data” in STAR Methods

Table S6. Additional data regarding the metabolomics experiments of plasma samples from sampling time point day 7, related to “data processing and integration of LC-MS acquired data” in STAR Methods

Table S7. Additional data regarding the metabolomics experiments of plasma samples from sampling time point day 28, related to “data processing and integration of LC-MS acquired data” in STAR Methods

Table S8. Additional data regarding the metabolomics experiments of plasma samples from sampling time point week 32, related to “data processing and integration of LC-MS acquired data” in STAR Methods

Table S9. Additional data regarding the metabolomics experiments of plasma samples from sampling time point term-equivalent age, related to “data processing and integration of LC-MS acquired data” in STAR Methods

Table S10. Target list of the suspect screening done on the untargeted metabolomics data, related to “data processing and integration of LC-MS acquired data” in STAR Methods

Table S11. List of reference standards used for testing system suitability, related to “QC & QA measurements for LC-MS methods” in STAR Methods

Table S12. List of analyte specific parameters of the LC-MS/MS method for investigating bile acids and bile acid amino acid conjugates, related to “targeted LC-MS/MS for bile acids and their conjugates” in STAR Methods

Document S2. Article plus supplemental information
