# Supplementary material for: Neuroactive metabolites and bile acids are altered in extremely premature infants with brain injury
Source: Cell Rep Med. 2024 Mar 22;5(4):101480. doi: 10.1016/j.xcrm.2024.101480 (PMC11031385; doi:10.1016/j.xcrm.2024.101480)
Supplement: Document S1. Figures S1–S5 [file mmc1.pdf]

Cell Reports Medicine, Volume *5*

Supplemental information

Neuroactive metabolites and bile acids are altered in extremely premature infants with brain injury

Manuel Pristner, Daniel Wasinger, David Seki, Katrin Klebermaß-Schrehof, Angelika Berger, David Berry, Lukas Wisgrill, and Benedikt Warth

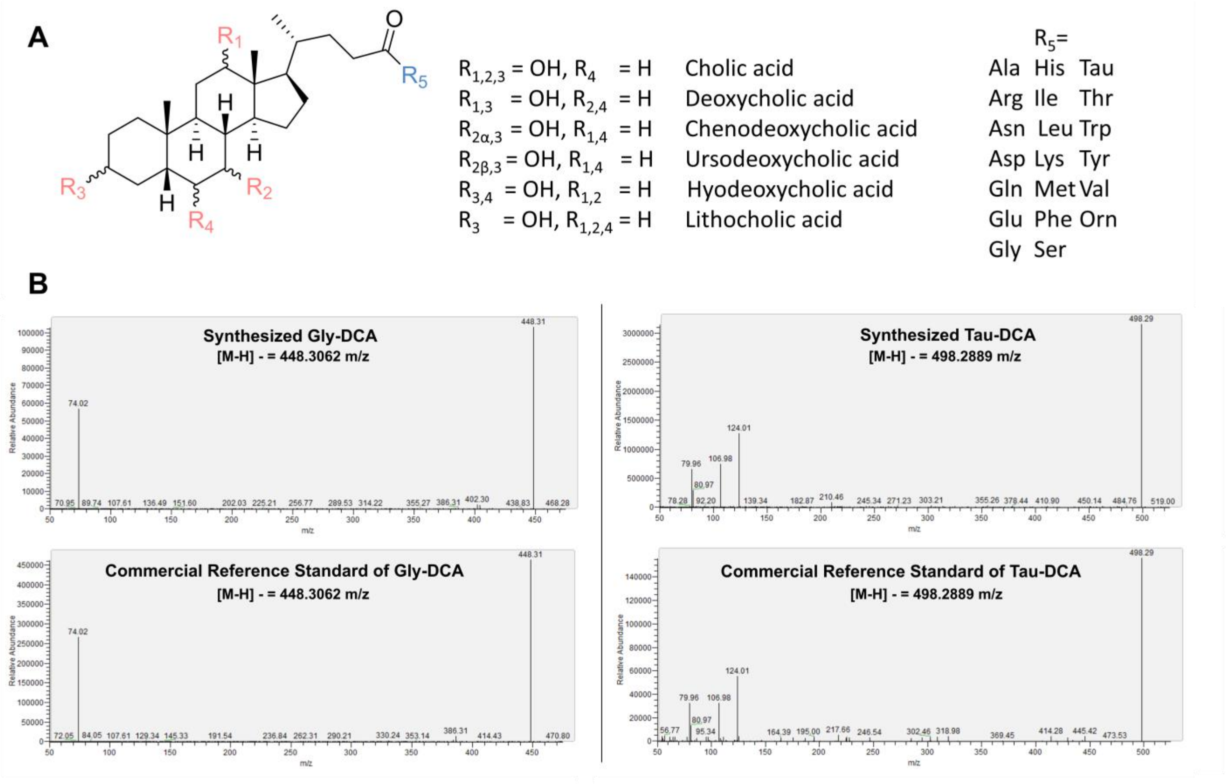

Figure S1: Synthesis of bile acid conjugates. Related to the 'Batch Synthesis of bile acid conjugates' section in the STAR Methods section. (A) Overview of synthesized bile acid amino acid and taurine conjugates described under the heading 'Batch Synthesis of bile acid conjugates' in the STAR METHODS section. **(B)** MS2 spectra of synthesized bile acid conjugates compared to commercial reference standards obtained from a reaction described under the heading 'Batch Synthesis of bile acid conjugates' in the STAR METHODS section.

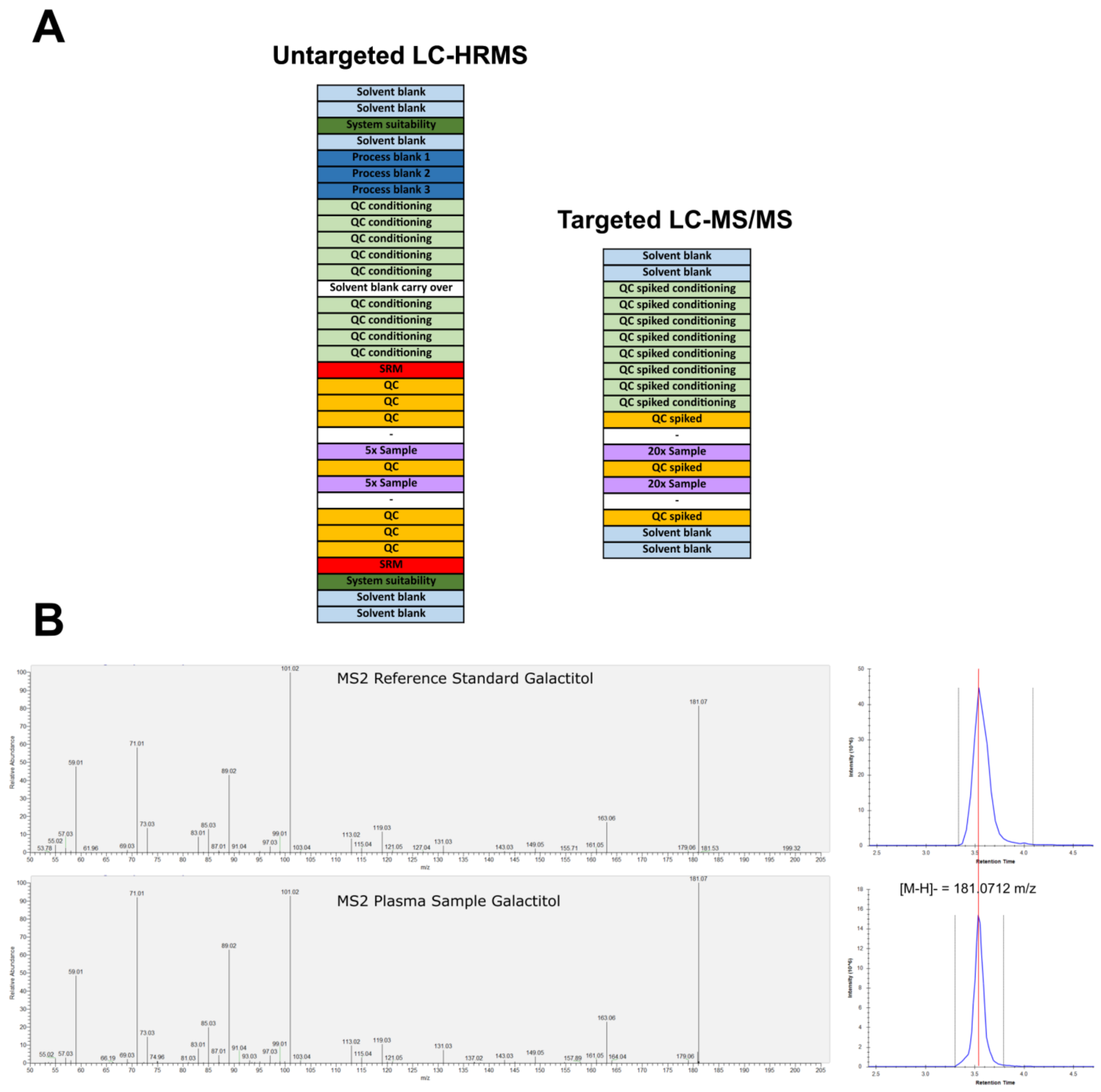

Figure S2: LC injection sequences and MS^2 spectra. Related to the 'QC & QA measurements for LC-MS methods' section in the STAR Methods. (A) Overview of the injection scheme of LC sequences for untargeted LC-HRMS and LC-MS/MS **(B)** MS^2 fragmentation pattern and extracted ion chromatogram of galactitol reference standard (2 µM) compared to feature found in biological sample and identified as galactitol.

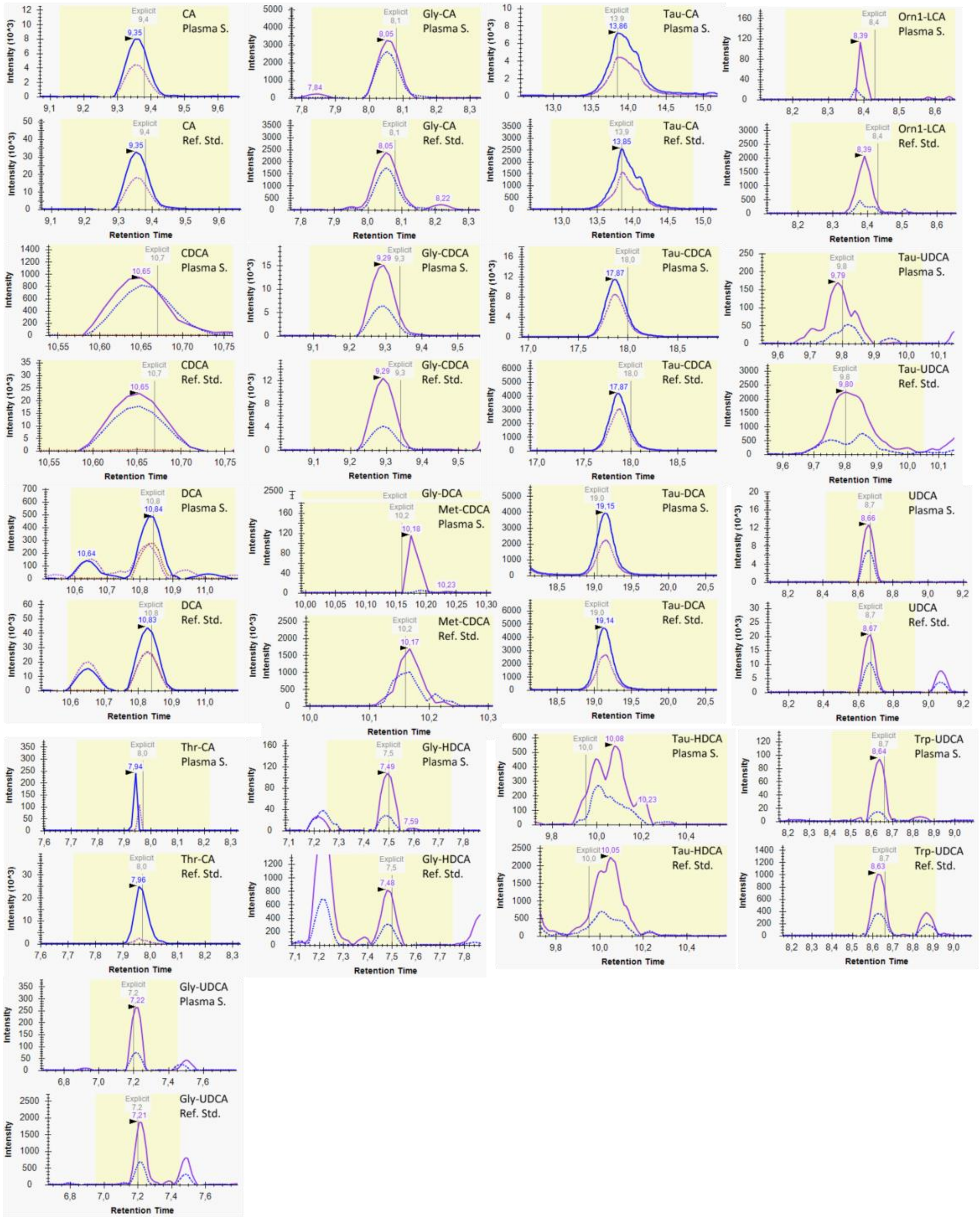

Figure S3: Extracted ion chromatograms of bile acids and bile acid conjugates detected in plasma. Related to the ‘Targeted LC-MS/MS for bile acids and their conjugates’ section in the STAR Methods. Extracted ion chromatograms of samples (Plasma S.) compared to the respective reference standards (ref.Std.).

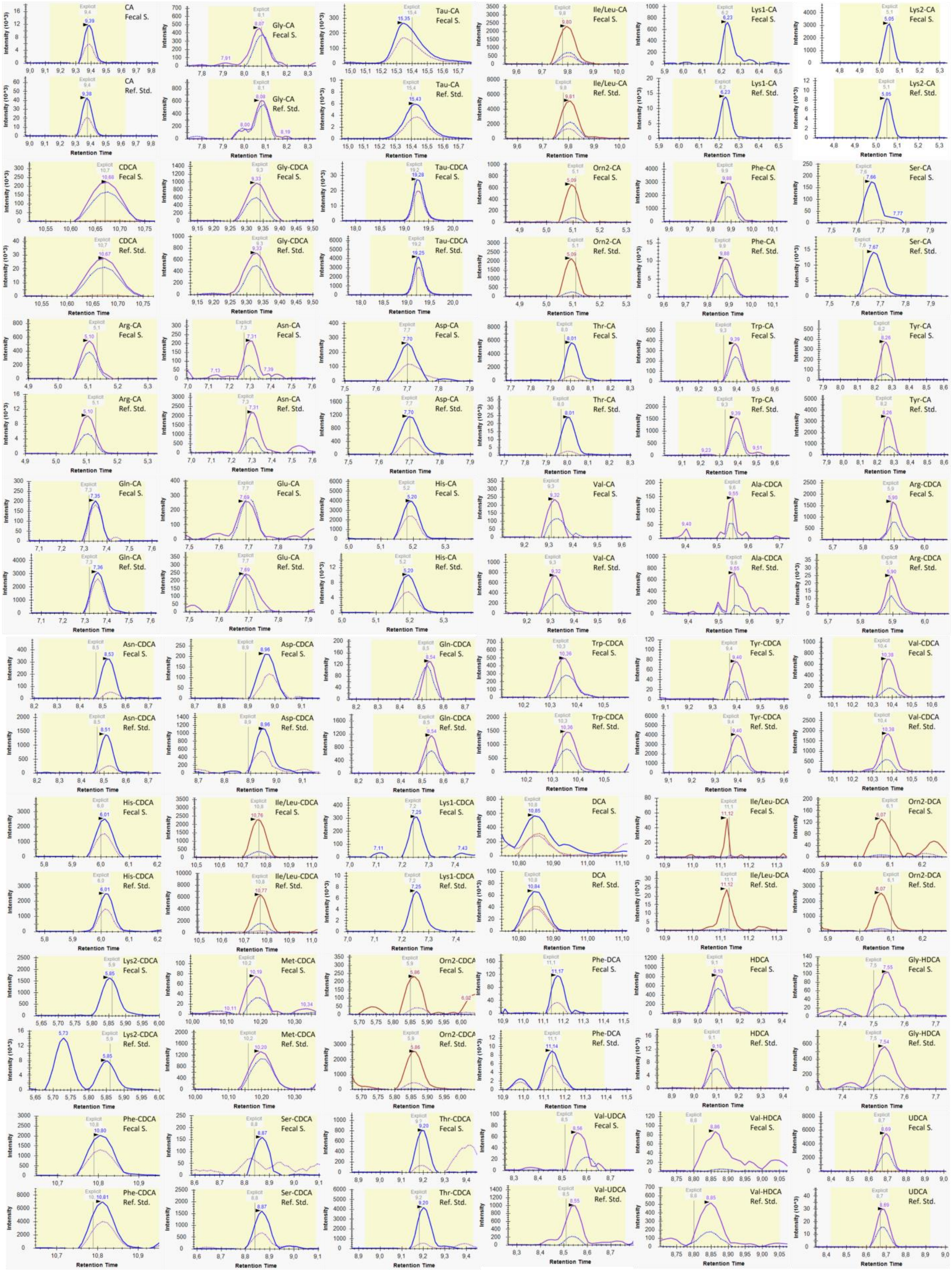

Figure S4: Extracted ion chromatograms of bile acids and bile acid conjugates detected in feces. Related to the 'Targeted LC-MS/MS for bile acids and their conjugates' section in the STAR Methods. Extracted ion chromatograms of samples (Fecal S.) compared to the respective reference standards (ref.Std.).

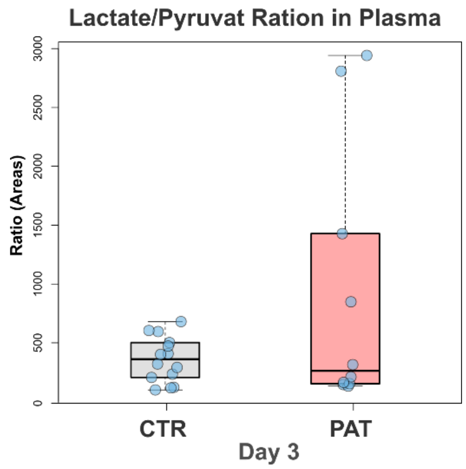

Figure S5: Ratio of lactate and pyruvate. Related to the Discussion section under the heading of 'Metabolic differentiation between experimental groups is already detectable in the earliest sample time point in both plasma and feces'. Ratio of lactate and pyruvate in plasma at sampling time point day 3 in the control and pathological group.
